# Radiofrequency ablation plays double role in immunosuppression and activation of PBMCs in recurrent hepatocellular carcinoma

**DOI:** 10.3389/fimmu.2024.1339213

**Published:** 2024-01-29

**Authors:** Yang Zhao, Tongwang Yang, Yabo Ouyang, Wei Rao, Kai Liu, Jiasheng Zheng, Fudong Lv, Ying Shi, Feng Wang, Dongjie Liu, Luxin Qiao, Zhenying Xia, Yushi Zhang, Dexi Chen, Wenjing Wang

**Affiliations:** ^1^ Department of Urology, Peking Union Medical College Hospital, Chinese Academy of Medical Science and Peking Union Medical College, Beijing, China; ^2^ The Affiliated Hospital of Qingdao University, Organ Transplantation Center, Qingdao, Shandong, China; ^3^ Beijing YouAn Hospital, Capital Medical University, Beijing Institute of Hepatology, Beijing, China; ^4^ Beijing Engineering Research Center for Precision Medicine and Transformation of Hepatitis and Liver Cancer, Beijing, China

**Keywords:** mass cytometry, radiofrequency ablation, recurrent hepatocellular carcinoma, PBMC, immunosuppresion

## Abstract

**Background:**

Radiofrequency ablation (RFA) is the primary curative treatment for hepatocellular carcinoma (HCC) patients who are not eligible for surgery. However, the effects of RFA on the global tumor immune response remain unclear.

**Method:**

In this study, we examined the phenotypic and functional changes in peripheral blood mononuclear cells (PBMCs) from recurrent HCC patients who had undergone two RFA treatments using mass cytometry and high-throughput mRNA assays.

**Results:**

We observed significant increase in monocytes and decrease in T cell subpopulations three days after the first RFA treatment and three days after the second RFA treatment. The down-regulation of GZMB, GZMH, GZMK, and CD8A, which are involved in the cytotoxic function of T cells, was observed following RFA. Furthermore, the population of CD8 effector and memory T cells (CD8 Teff and CD8 Tem) significantly decreased after RFA. The expression of CD5 and CD161 in various T cell subpopulations also showed significant reductions. Additionally, elevated secretion of VEGF was observed in monocytes, B cells, regulatory T cells (Tregs), and CD4 naive T cells.

**Conclusion:**

In recurrent HCC patients, serum components derived from radiofrequency therapy can enhance the antigen-presenting capacity of monocytes. However, they also inhibit the anti-cancer immune response by reducing the population of CD8 effector and memory T cells and suppressing the activation of T cells, as well as down-regulating the expression of CD161 and CD5 in various T cell subpopulations. These tumor-derived components also contribute to an immunosuppressive microenvironment by promoting the secretion of VEGF in monocytes, Tregs, B cells, and CD4 naive T cells.

## Introduction

Hepatocellular carcinoma is one of the most common malignancies, ranking as the third most lethal malignancy worldwide (1, 2). Following primary treatment, a large proportion of patients suffered cancer recurrence. For HCC patients ineligible for surgical resection or liver transplantation, radiofrequency ablation (RFA) has emerged as a frequently employed curative approach. Other than direct damage to tumor tissue, RFA also has the potential to influence the overall immune response (3), which may lead to a durable anti-cancer immune response and improved tumor control. Nevertheless, this alone does not guarantee complete remission. Approximately 13.3% patients receiving RFA experienced local recurrence, while 45% suffered from distant recurrence within 3 years (4). Notably, inflammation induced by incomplete radiofrequency ablation accelerates tumor progression and hinders PD-1 immunotherapy. Therefore, the detailed understanding of the effect of RFA on immune cells in patients with recurrent HCC is urgently needed for further clinical trials.

Peripheral blood mononuclear cells (PBMC) are believed to be the origin of the tumor infiltrating immune cells. Given the challenges in tumor sample acquisition in RFA, PBMCs are considered to be the optimal target to analyze the overall immune status following RFA. Notably, PBMC components were reported to change remarkably after RFA and can serve as biomarkers for the effectiveness of RFA treatment (4, 5). However, these clinical studies could study only a few subtypes of the immune cells because of the limited number of signal channels of flow cytometry. Mass cytometry, also known as cytometry by time-of-flight (CyTOF), uniquely combines metal-labeling of antibodies with mass spectrometry to enable high-dimensional measurement of the characteristics of individual cells (6). This technology enables us to analyze immune cell subsets and the expression of functional molecules at the single-cell level.

In this study, we used mass cytometry to comprehensively characterize the phenotypic and functional alterations of PBMCs from the patients with recurrent HCC who received RFA treatment, aiming to unveil the dynamic landscape of the anti-cancer immune response following both the primary and secondary RFA. Also, the immune–related mRNA profiles of the PBMC were analyzed using a high throughput RNA assay system, identifying the down-regulated expression of markers for a set of cytotoxic function-related genes after RFA. These findings were further validated by mass cytometry analysis on health donor-derived PBMC treated with serum from HCC patients before and after RFA. This research offers valuable insights for future investigations on the potential combination of immunopotentiating therapy with RFA in the treatment of HCC.

## Materials and methods

### Patients

In this study, blood samples were obtained from 21 recurrent HCC patients who received primary and secondary RFA therapy in YouAn Hospital from January 2011 to January 2015. The primary eligibility criteria included patients with primary HCC, who would undergo RFA with curative intent. The diagnosis of HCC was preferably histologically confirmed. In cases when tumor biopsy results were unavailable, diagnosis was established by contrast-enhanced CT or MRI. Treatment selection in each patient was decided by the clinical doctors guided by the NCCN and Chinese HCC treatment guidelines. Blood samples were collected before RFA treatment and three days after RFA and stored at. -80°C. Follow-up assessments and imaging examinations were conducted periodically for a minimum of 3 years. Patients who had HCC recurrence diagnosed by imaging examinations and received second RFA were finally enrolled. Patient characteristics and disease classification are shown in **Table 1**. All patients provided written informed consent and this study was approved by the Medical Ethics Committee of YouAn Hospital.

**Table 1:** The Baseline Clinical and Pathological characters of the patients.

ID	Age	Gender	Hepatitis	Pathological results									
				Pathological type	Degree of differentiation;	HBS Ag	HBC Ag	Hepa	CK19	CD34	P53	GPC3	Ki67
1	48	Male	HBV	HCC	NA	+	–	+	–	+	–	+	+
2	46	Male	HBV	HCC	Moderate	+	–	+	–	+	–	+	+
3	72	Male	HBV	HCC	Moderate	+++	–	+++	–	+	+	+++	+
4	63	Male	HBV	HCC	Moderate	+	–	+	–	–	+	–	+
5	52	Male	HBV	HCC	Low	+	–	+	–	+	+	+	+
6	55	Male	HBV	HCC	High	+	–	+	–	+	–	–	–
7	68	Male	HBV	HCC	High	+	–	+++	–	++	–	++	+
8	61	Male	HBV	HCC	Moderate	++	–	+++	–	–	+	–	+
9	67	Male	No	HCC	Moderate	–	–	+++	–	–	+	+	++
10	47	Male	No	HCC	Moderate	–	–	+	–	–	+	+	+
11	56	Male	No	HCC	Moderate	–	–	+	–	+	+++	–	++
12	40	Male	No	HCC	Moderate	–	–	+	–	+	–	–	+
13	68	Male	HBV	NA	NA	NA	NA	NA	NA	NA	NA	NA	NA
14	68	Male	No	HCC	Moderate	–	–	+++	–	+	++	±	+
15	44	Male	No	HCC	Low	–	–	+	–	–	+++	+++	+
16	55	Male	HBV	HCC	High	+	–	+	–	+	–	–	–
17	56	Male	HBV	HCC	Moderate	–	–	+	–	+	++	+++	+
18	72	Male	No	HCC	Moderate-Low	–	–	+	–	+	+	±	+
19	47	Male	No	HCC	Moderate	–	–	+	±	+	–	+	+
20	53	Male	No	HCC	High	+	–	+++	–	++	–	++	+
21	56	Male	HBV	HCC	High	+	–	+	–	+	–	–	–

Patient 13 was diagnosed by CT and ultrasonography examinations, while other patients were diagnosed by pathological examinations. HCC, Hepatocellular carcinom; HBV, Hepatitis B virus; HBSAg, Hepatitis B surface antigen; HBC Ag, Hepatitis C surface antigen; CK19, Cytokeratin 19; CD34, Cluster of differentiation 34; GPC3, glypican; NA, not available.

-: Negative;+: weakly positive ++ :moderate positive +++: strongly positive.

### PBMC isolation and storage

Venous blood samples were obtained on the day before the first RFA treatment, as well as 3 days after the first treatment, the day before the second RFA treatment, and 3 days after the second treatment. PBMCs were separated from whole blood using Human Peripheral Blood Lymphocytes Separation Medium (#P8610, Solarbio, Beijing, China) by means of density gradient centrifugation following the instruction of the kit. The isolated PBMCs were stored at -80°C until the mass cytometry or mRNA examination.

### *In vitro* PBMC stimulation

Serum samples was collected from patients who received RFA before and after RFA treatment, inactivated at 56°C for 30mins, and then used for PBMC culture. For removal of the big molecules, the supernatant of serum was transferred to the centrifuge tube, centrifuged at 4500rpm for 5 min, the serum in the centrifuge tube containing big molecules was removed and the serum in the bottom was collected and filtered by 0.22um filter mesh, then the serum was used for PBMC culture. PBMCs from healthy donor were cultured overnight in 6-well plates at a concentration of 1 × 10^6^cells/well, followed by exposure to prepared HCC patient’s serum (preparation was described above). After 48 hours incubation, PBMCs were stimulated with leukocyte activation cocktail (plus protein transport inhibitors, BD Biosciences, USA) for 4 hours before mass cytometry examination.

### Mass cytometry

Pre-conjugated antibodies panels (Maxpar human T cell phenotyping panel kit and expansion panel kit) were purchased from Fluidigm Company. Samples were rapidly rewarmed, then washed and stained with cell surface antibodies for 30 minutes on ice. After that, PBMCs were permeabilized at 4°C overnight and stained with intracellular antibodies for 30 minutes on ice. The antibody-labeled PBMCs were washed and incubated in 0.125nM Ir intercalator (Fluidigm) diluted in PBS containing 2% formaldehyde, and stored at 4°C until the mass cytometry examination.Before acquisition, samples were washed with de-ionized water and then resuspended at a concentration of 1×10^6^ cells/ml with deionized water containing a 1/20 dilution of EQ 4 Element Beads. PBMCs were then examined by a CyTOF2 mass cytometry (Fluidigm DVS Sciences Inc., Toronto, ON, Canada).

### High-throughput mRNA analysis

Total RNA was extracted from PBMCs using the QIAamp RNA Mini Kit following the manufacturer’s instructions. The random primer was used to synthesis cDNA following the standard protocol of SuperScript III RT kit (Invitrogen). The synthesized cDNA was added to 1.5ml tube added with 190ul MILIQ H2O. The diluted cDNA was added with 200ul saturated phenolic chloroform mixture in the ice for 10min and centrifuged at 12000g for 10min. The upper aqueous phase was collected into a new 1.5ml tube added with 2ug glycogen and 500ul ethanol. After stored at -80 degree refrigerator for 8h, the sample was centrifuged at 14000g for 30min. The pellet was wash with 1ml 70% ethanol and centrifuged at 10000g for 5min. The cDNA was dissolved in 10ul H2O. cDNA from PBMCs was screened for expression of housekeeping genes ACTB and GAPDH and then were analyzed with TaqMan Universal PCR Master Mix (Applied Biosystems, Foster City, CA, USA), 20X GE Sample Loading Reagent (Fluidigm, South San Francisco, CA, USA), 2X Assay Loading Reagent (Fluidigm, South San Francisco, CA, USA) and individual TaqMan Gene Expression Assay using 96.96 Dynamic Array™ IFC (Fluidigm, South San Francisco, CA, USA) on a BioMark HD System (Fluidigm, South San Francisco, CA, USA). Ct (threshold cycle) values were processed and analyzed using the BioMark Real-Time PCR Analysis software (Fluidigm South San Francisco, CA, USA).

### Statistical analysis

Mass Cytometry data were firstly normalized using EQ Four Element Calibration Beads (EQ Beads, 201078, Fluidigm) according to manufacturer’s instructions, then the cell debris were removed according the 191Ir and 193Ir channel. Doublets were removed according to the Even Length. Furthermore, CD45 gate were used to isolate all the blood leukocyte, then a clustering panel including CD3, CD4, CD8A, CCR4, CCR5, CCR7, HLA-DR, CD11A, CD16, CD25, CD44, CD45RA, CD45RO and CD127 were used to manually differentiate subpopulations of the blood leukocytes. Meanwhile, blood leukocytes were also clustered into 200 nodes using the SPADE analysis with a 20% down-sampling rate. Then repeated measurements ANOVA were performed to analyze the difference of cell count and functional marker expression of the subpopulations between the four time points. Paired t test were used to analyze the difference of mRNA expression, cell count and protein expression of the subpopulations if only samples of two time points are available. To further explore the alteration in subpopulations and functions of T cells, a viSNE analysis was performed using the panel including CD4, CD8A, CCR4, CCR5, CCR7, CD45RA, CD45RO, PD-1, LAG3, TIM3, CTLA4, OX40, 4-1BB, ICOS, CD28, FAS, and CXCR3. To validate the findings of high-throughput mRNA assay, a additional antibody panel were used in the mass cytometry analysis. In this panel, TNFα, IFNγ, TGFβ, pNFkB_P65, CD86, GM-CSF, IL-10, CD284, IL-2, MHCII, CD206 antibodies were added, while functional markers which have been already detected in the first antibody panel, including LAG3, TIM3, CTLA4, OX40,4-1BB, ICOS, CD28, FAS, were removed.

Bead-based normalization was performed using the CyTOF software. Manual gating, SPADE analysis and vi-SNE analysis were performed using the Cytobank software (https://premium.cytobank.org). Repeated measurements ANOVA were performed using R software and its CAR package. Bubble plot, box plot and heat maps were prepared using R software (version 3.3), pheatmap and ggplot2 package and Graphpad Prism 5 software. KEGG pathway enrichment were performed using the DAVID software (version 6.8, https://david.ncifcrf.gov/). Protein-protein interaction were analyzed using the STRING software (version 10.5, https://string-db.org/) and cytoscape software (version 3.4.0).

## Results

### Patients

In this study, 24 recurrent HCC patients were enrolled, and all of them received second RFA. Three of them were excluded because of loss of follow-up after the second RFA. The number of isolated PBMC of four patient was insufficient to complete mass cytometry. The clinical characteristics of the enrolled patients were shown in **Table 1**. Blood samples were collected at four time points: the time before the first RFA, 3 days after the first RFA, before the second RFA, and 3 days after the second RFA. Then mass cytometry was performed on the PBMC samples from 17 patients. High-throughput mRNA analysis was performed on the pre-RFA and post-RFA serum collected from 4 patients.

### Manual gating and SPADE analysis

**Figure 1:**
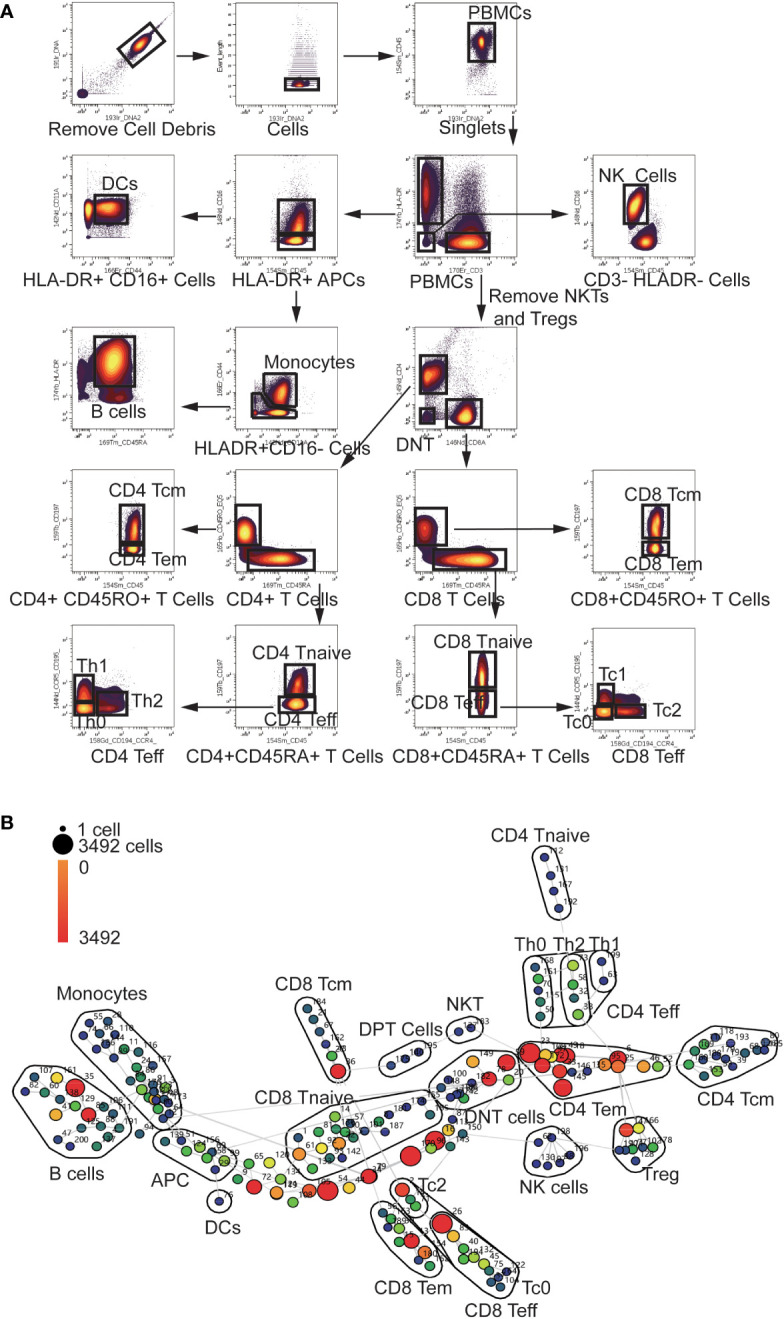
Gating Strategy and annotation of SPADE nodes **(A)** Gating Strategy: After Normalization, cell debris were removed according to the Ir191 and Ir193 channels. Then data of singlets were collected using event length channel. CD45+ PBMCs were divided into HLA-DR+ APCs, CD3+ T cells and HLA-DR-CD3- cells. HLADR+ APCs were then divided into Monocytes, DCs, and B cells using the CD16, CD11a, CD44 and CD45RA channels. NKTs and Tregs were removed from CD3+ T cells using the CD16, CD25, and CD127 channels (not shown in the scheme).The T cells were then divided into CD4 and CD8 T cells using the CD4 and CD8 channels. CD4 and CD8 cells were divided into Tcm Tem Tnaive and Teff cells using the CD45RA, CD45RO, and CCR7 channels. CD4 and CD8 Teff cells were further divided into Th0, Th1, Th2, Tc0, Tc1 and Tc2 using the CCR4 and CCR5 channels. **(B)** Annotation of SPADE nodes: CD45+ PBMCs were divided into 200 nodes using the clustering antibody panel and annotated according to the expression of clustering markers of every node. The node id is marked aside the nodes and the cell count of every node is indicated with the color of the node. Each node represents a cluster of cells which has similar marker expression. The detailed marker expression of every node was shown in **Supplementary Figure S2**. The nodes with similar marker expression pattern were lined with grey lines.

To explore the multi-dimension mass cytometry data, we analyzed CD45+ blood leukocytes through the implementation of manual gating and SPADE analysis. The gating strategy employed were displayed in **Figure 1A**. The significant altered PBMC subpopulations identified by manual gating were shown in **Supplementary Figure S1**.

In the SPADE analysis, numbers of clusters were set to 200 to avoid omission of rare and small cell subpopulation. These clusters were then annotated according to the median expression of cell markers. The analysis revealed a diverse range of clusters, including 43 CD4 T cell clusters, 50 CD8 T cell clusters, 10 Treg clusters, 6 NK clusters, 2 NKT clusters, 26 Monocyte clusters, 3 DPT (double positive T cells) clusters, 11 DNT (double negative T cell) clusters, 18 B cell clusters and one DC (Dendritic Cell) clusters (**Figure 1B**). Eight HLA-DR+CD3-CD16-CD45RA-CD44^low^CD11A^low^ antigen-presenting cells (APC) clusters and 22 CD3- HLA-DR-CD16- clusters were not recognized by our panel. The detailed marker expression of every SPADE node was shown in **Supplementary Figure S2**.

### The alteration of APCs after RFA treatment

APCs are major players in initiation of anti-tumor immune response. Among CD45+ cells, HLA-DR+ APCs constituted a substantial proportion, accounting for 25.11% (25 and 75 percentiles: 17.88-39.73%). Within the HLA-DR+ APC population, monocytes constituted 13.23% (8.18-25.8%), DCs accounted for 0.09% (0.03-0.17%), and B cells represented 4.02% (2.8-7.84%), and a big proportion of APCs cannot be recognized by our panel, which took 4.18% (2.08-7.03%) of all the CD45+ cells.

Following RFA treatment, there was a rapid and significant increase in the percentage of monocytes, which subsequently returned to normal levels prior to the second RFA (P=0.026, **Supplementary Figure S1A**). A sustained reduction of B cell count was observed after the first RFA (P=0.021, **Supplementary Figure S1B**), and the B cell count remained at a low level before and after the secondary RFA. The B cell count was also reduced in 13 clusters defined by SPADE analysis.

### Populational and functional landscape of CD3+ T cells

Following the first RFA treatment, there was a significant reduction in the percentage of CD4+ T cells within the CD45+ cells, and this reduction was observed again after the second RFA (P=0.024, **Supplementary Figure S1C**). CD8+ T cells showed the same alteration as that of CD4+ cells (P=0.03, **Supplementary Figure S1D**). We then studied the subpopulation of CD4+ and CD8+ T cells using SPADE analysis. Primary SPADE analysis yielded 45 CD4 T cell clusters, 49 CD8 T cell clusters, 3 DPT clusters and 11 DNT clusters. Among the CD4^+^ T cell clusters, 10 clusters represented CD45RA^high^CD45RO^low^CCR7^-^ CD4 Teff (T effector) clusters, 4 clusters represented CD45RA^high^CD45RO^low^CCR7^+^ CD4 T naïve cells, 15 clusters represented CD45RA^low^ CD45RO^high^CCR7^-^ CD4 Tem (T effector memory) cells and 16 clusters represented CD45RA^low^ CD45RO^high^CCR7^+^ CD4 Tcm (T central memory) cells. Among the CD8 T cell clusters, 14 clusters represented CD45RA^high^CD45RO^low^CCR7^-^ CD8 Teff cells, 20 clusters represented CD45RA^high^CD45RO^low^CCR7^+^ CD8 T naïve cells, 9 clusters represented CD45RA^low^ CD45RO^high^CCR7^-^ CD8 Tem cells and 6 clusters represented CD45RA^low^ CD45RO^high^CCR7^+^ CD8 Tcm cells.

**Figure 2:**
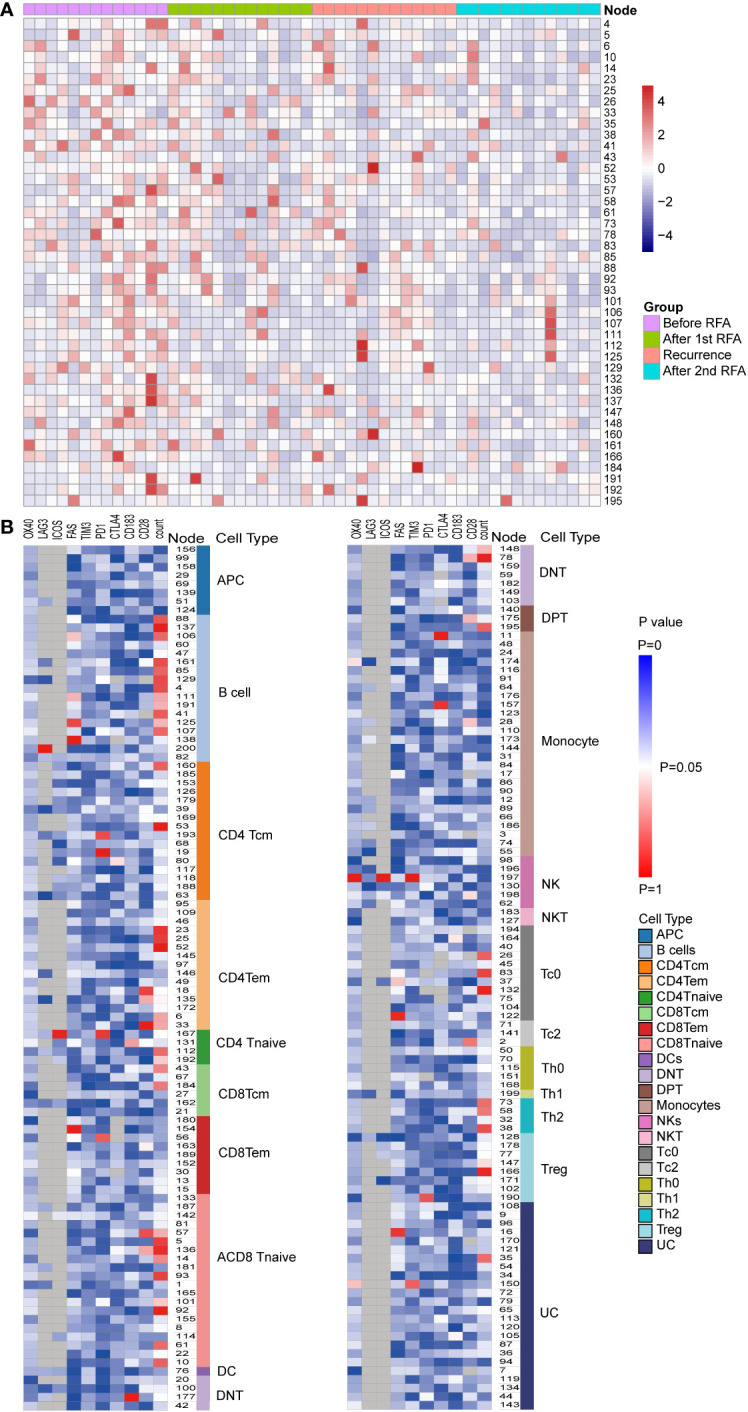
Populational and functional alteration of SPADE nodes. **(A)** Population distributions of SPADE nodes with significant population alteration identified by repeated measurement ANOVA (P<0.05) were displayed using heatmap. The nodes were colored according to the normalized Z score of the population of nodes, which were calculated using the percent of node cell counts in the CD45 PBMCs. The detailed population data could be found in **Supplementary Table S1**. **(B)** Significance of alteration in population and expression of functional markers, including PD-1, LAG3, TIM3, CTLA4, OX40,4-1BB, ICOS, CD28, FAS, and CXCR3, were displayed in heat map. Each heat map unit was colored according to the repeated measurement ANOVA P value for the four time points.

Furthermore, we annotated the T effector clusters according to the expression of the surface marker CCR4, CCR5, and CCR7. Within the CD4 T effector cell clusters, five clusters were identified as CCR4-CCR5-CCR7- Th0 cells, one cluster was identified as CCR4-CCR5+CCR7- Th1 cell and four clusters were identified as CCR4+CCR5-CCR7- Th2 cells. Within the CD8 T effector cell clusters, 11 clusters were identified as CCR4-CCR5-CCR7- Tc0 cells and 3 clusters were identified as CCR4+CCR5-CCR7- Tc2 clusters. (**Figures 2A, B**, **Supplementary Table S1**).

**Figure 3:**
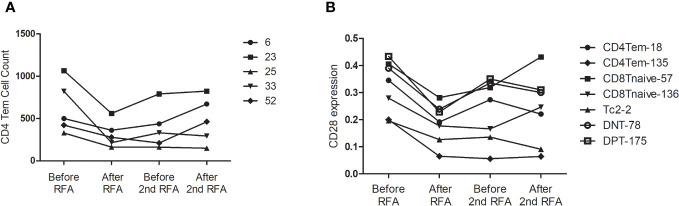
**(A)** CD4Tem nodes with significant populational alterations. CD4Tcm nodes with significant populational changes among the four time points (P<0.05) were shown. All these clusters showed reduced population after the first RFA, and after the second RFA, the number of Tem clusters showed no change or moderate increase. **(B)** T cell nodes with significant alterations in CD28 expression. Nodes with significant changes in CD28 expression among the four time points (P<0.05) were shown, including two CD4 Tem cluster (Node 18 and 135), two CD8 T naïve clusters (Node 57 and 136), one DPT cluster (Node 175), and one DNT cluster (Node 78). All these clusters showed decreased CD28 expression after the first RFA. After the second RFA, CD8 Tnaive clusters showed increased CD28 expression, the other 5 clusters showed decreased CD28 expression.

We then analyzed the change in cell count of T cells at the four designated time points. Six CD4 Tem clusters showed a reduced population after the first RFA. After the second RFA, the number of Tem clusters showed no change or moderate increase (P<0.05 for all the six Tem clusters) (**Figure 3A**). Two CD4 Tcm clusters showed reduced populations after the first RFA, which were then restored to a lower level before the second RFA started (P=0.002, 0.021, respectively, **Figure 3A**). Two CD4 T naive clusters showed increased cell counts at time points 3 and 4 (P=0.023, 0.042, respectively, **Figure 3A**). With respect to CD4 effector T cells, 3 Th2 clusters showed significant population alteration across the 4 time points. All the Th2 clusters showed a more than 3-fold decrease after the first RFA. Cluster 38 and 58 had a slight decrease while Node 73 showed a significant increase after the second RFA (P=0.017, 0.026, 0.024, respectively, **Supplementary Table S1**). Three Tc0 clusters showed major or moderate decrease after the first and second RFA (P=0.022, 0.015, 0.006, respectively) (**Supplementary Table S1**).

Next, we focused on the expression of co-stimulatory molecule of T cells. Significant alterations of CD28 expression were found in 7 T cells clusters, including two CD4 Tem clusters, two CD8 T naïve clusters, one DPT cluster, and one DNT cluster (P<0.05 **Figure 3B**). However, following the first RFA, these seven clusters displayed decreased CD28 expression. Interestingly, 2 CD8 T Naïve clusters showed increased CD28 expression after the second RFA, while the other 5 clusters showed decreased CD28 expression again after the second RFA (**Figure 3B**). Cluster 167, identified as a CD4 Tnaive cluster, showed significant ICOS expression after the first RFA, and remained in a high ICOS expression level before and after the second RFA (P=0.049). A reduction in PD-1 expression of cluster 167, a CD4 Tnaive cluster, and cluster 193, a CD4 Tcm cluster, was found after the first and second RFA (P=0.022). No significant alterations of 4-IBB, OX-40 were found in CD3+ T cell clusters (**Supplementary Table S2**).

Furthermore, we investigated the expression of inhibitory molecules, and expression of PD-1 were altered in Node 19, 56, 167,190 and 193, which were identified as CD4Tcm, CD8 Tem, CD4 Tnaive, Treg and CD4 Tcm, respectively (**Supplementary Table S2**). An elevated PD-1 expression after the first and second RFA were found in Node 19 and 56. While no significant alterations were found in the expressions of TIM3, CTLA-4 and LAG-3.

Furthermore, a viSNE analysis was performed in 5 patients with more than 10 thousand CD3+ T cells at all four time points to explore the functional subpopulations of T cells (**Supplementary Figure S3**), We found that CD3+ T cells of patient 9 showed remarkable CTLA-4 expression and the expression of CTLA-4 was greatly reduced, accompanied with an increase of CD28 expression at time points 3 and 4 (**Supplementary Figure S4**). In the other four patients, CTLA-4 was mainly expressed in DPT cells.

### NK cells showed down-regulated ICOS and OX40 expression after the first RFA

NKs cells are crucial components in the anti-tumor immune response after RFA, which could eradicate the tumor cells directly or induce the apoptosis of tumor cells by secreting IFN-α and TNF-α (7). Notably,we examined the cell count of NK cells and NKT cells and found no significant alterations across the four time points.

### High-throughput mRNA assays revealed immunosuppressant mRNA profile after RFA

**Figure 4:**
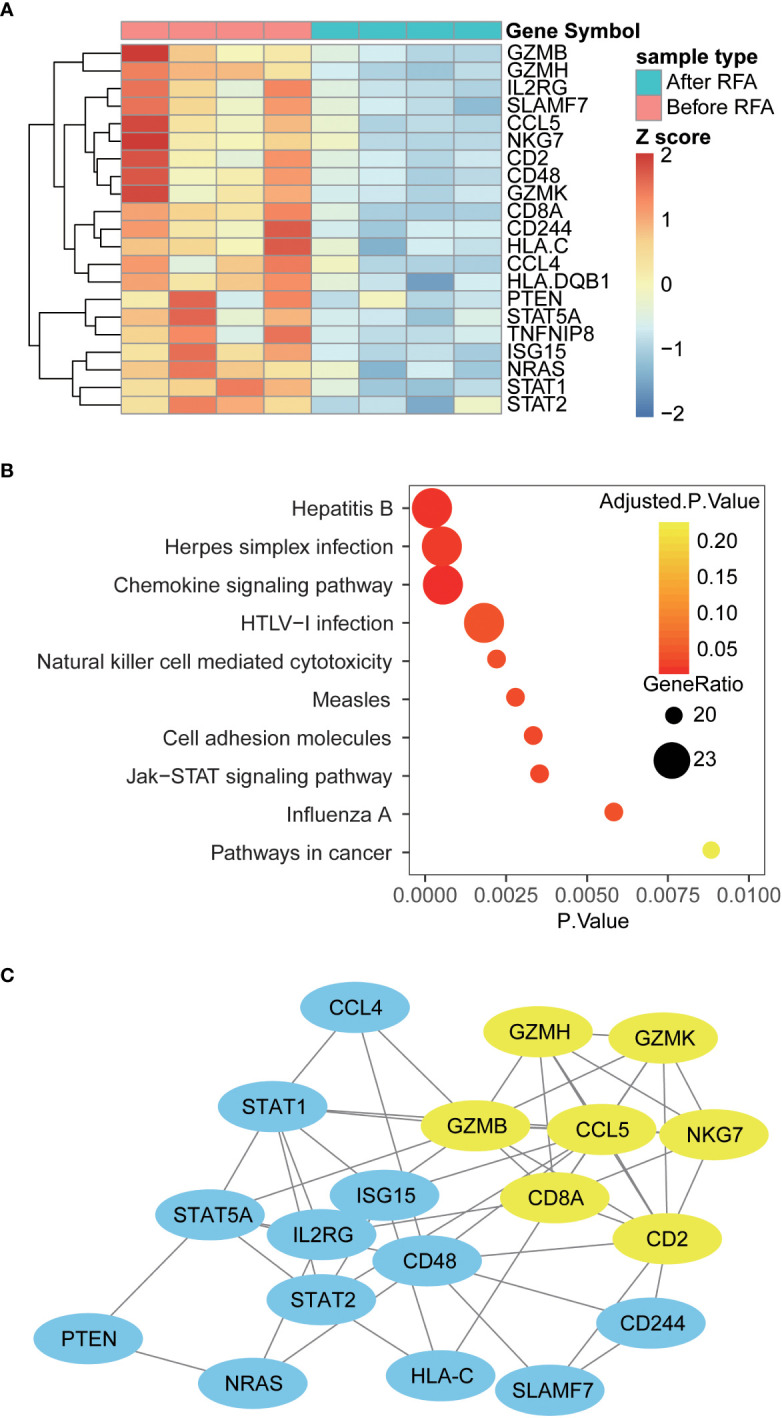
Differently expressed genes, altered pathways and PPI network in PBMC before and after RFA **(A)** Heat map of Differently expressed genes. Genes with significantly altered expression are displayed in heat map. Overall, 21 genes were differently expressed in PBMC before and after RFA(P<0.05). **(B)** Top 10 enriched KEGG pathways. Enrichment analysis was performed on genes with significant mRNA changes between the two time points using DAVID software. The top 10 enriched pathways were displayed using the bubble plot. The x axis represents the P value of the pathways; the size of the dots represents the generatio of the altered genes versus all genes in the pathways; and the color of the dots represents the Benjamin adjusted P value of the pathways. **(C)** Protein-protein interaction network of the Differently expressed genes. PPI networks were constructed using the STRING software, and the links between the nodes represents the possible protein interaction identified by the STRING software. The yellow nodes represent the sub-network identified by the MCODE plug-in of cytoscape software.

**Figure 5:**
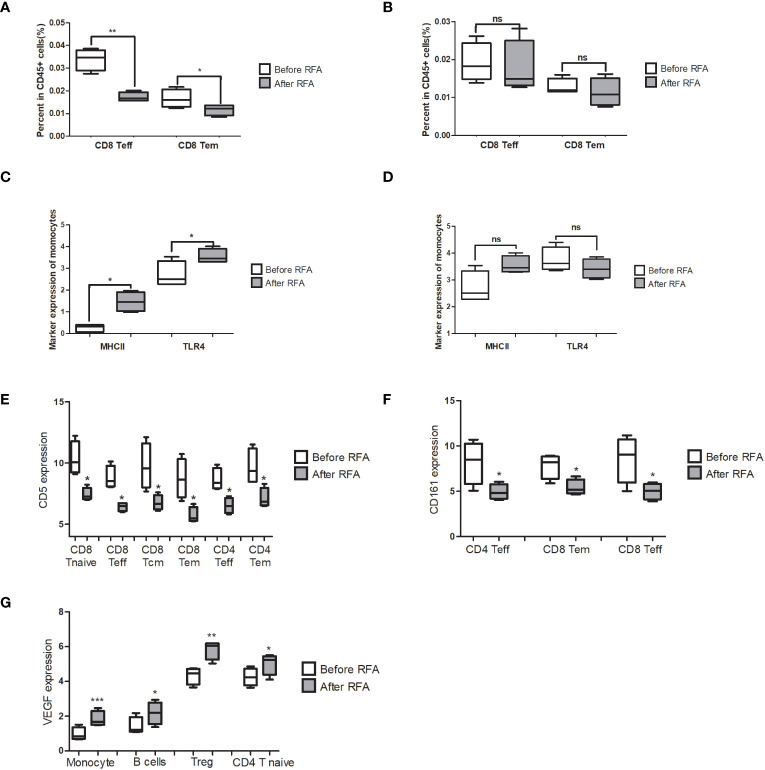
Significantly changed populations or biomarkers in the PBMC treated with before or after RFA serum from HCC patients. **(A)** Population of CD8 Teff and CD8 Tem significantly reduced in the post-RFA serum-treated PBMC (P = 0.002 and 0.04, respectively). **(B)** When >10KD components were removed from the serum, no significant alteration was found in the population of CD8 Teff and CD8 Tem between the two groups (P=0.771 and 0.466, respectively). **(C)** MHCII and TLR4 expression of monocytes significantly elevated in the after RFA group (P = 0.033 and 0.022, respectively). **(D)** When >10KD components were removed from the serum, no significant alteration was found in the MHCII and TLR4 expression of monocytes between the two groups (P=0.632 and 0.433, respectively). **(E)** In the PBMC treated with post-RFA serum, a significant reduction of CD5 expression in a wide span of T cells was found, including CD8 Tcm, CD8 Teff CD8 Tem, CD8 T Naïve, CD4 Teff and CD4 Tem (P=0.041, 0.028, 0.026 0.023, 0.046 and 0.042, respectively). **(F)** In the PBMC treated with post-RFA serum, expression of CD161 in CD4 Teff, CD8 Tem, and CD8 Teff significantly reduced, compared with the PBMC treated with pre-RFA serum (P=0.025, 0.025 and 0.038, respectively). **(G)** In the PBMC treated with post-RFA serum, the expression of VEGF significantly increased in monocytes, B cells, Tregs, and CD4 T Naïve cells (P = 0.0003, 0.024, 0.002 and 0.038, respectively). NA: Not available * P<0.05, ** P<0.01, *** P<0.001, ns, not significant

To extend our knowledge of impact of RFA on the functional alteration of PBMC, we proceeded to examine the mRNA profile of PBMC using a high-throughput gene assay system. The expression of a panel of 148 immune-related genes was examined in samples from four HCC patients. Overall, we discovered 21 differently expressed genes in PBMC collected after RFA, compared with the PBMC collected before RFA (**Figure 4A**). Interestingly, CD2 and CD8A, which is known as T cell-specific surface markers, and GZMB, GZMH, GZMK, and NKG7, which is closely related to the cytotoxic function of T cells, showed decreased mRNA expression in PBMC after RFA. Also, the expression of chemokine CCL4 and CCL5 significant decreased (**Figure 5A**). Furthermore, the protein-protein interaction analysis again revealed an inhibited PPI network relating to the T cell function (**Figure 4B**). Further functional pathway analysis using DAVID software demonstrated that the Natural killer cell mediated cytotoxicity, and the JAK-STAT signaling pathway were significantly inhibited after RFA (**Figure 4C**, **Supplementary Table S3**). These data again showed an immunosuppressant effect of RFA on PBMC, especially on T cells.

### *In vitro* stimulating of PBMC by post-RFA serum of HCC patients

We have demonstrated above that RFA could cause the population and gene signature of PBMC to change towards an immunosuppressant status. Given that the major mechanism of RFA involves the direct destruction of tumor tissue and cause the necrosis in the remaining cancer cells, we hypothesized that the components released by the necrosis tumor tissue is the main cause of the immunosuppression effect of RFA. We then treated the PBMC from a health donor with before-RFA and post-RFA serum from four HCC patients to validate the immunosuppression effect of post-RFA serum. We found that after treated by post-RFA serum for 48 hours, the percentage of CD8 Teff and CD8 Tem in CD45+ cells significantly decreased (**Figure 5A**; P= 0.002 and 0.040, respectively).

In terms of functional markers, the expression of MHCII and TLR4 of monocytes significantly elevated compared to the PBMC treated with post-RFA serum, indicating an enhanced antigen-presenting function of monocytes (**Figure 5C**; P= 0.033 and 0.022, respectively). However, no significant changes were found in GM-CSF, TNFα, IFNγ and p-NFκB expression of monocytes. Instead, the secretion of VEGF, which is increased in tumor-associated macrophages, significantly increased in monocytes (**Figure 5G**, P<0.001). Increased VEGF expression was also found in B cell, Treg, and CD4 T Naïve (**Figure 5G** P= 0.024, 0.002 and 0.038, respectively). Meanwhile, a significant reduction of CD5 expression in a wide span of T cells was found, including CD8 Tcm, CD8 Teff, CD8 Tem, CD8 T Naïve, CD4 Teff and CD4 Tem (**Figure 5E**; P=0.041, 0.028, 0.026 0.023, 0.046 and 0.042, respectively), as well as a significant reduction of CD161 expression in CD4 Teff, CD8 Tem, and CD8 Teff (**Figure 5F**; P=0.025, 0.025 and 0.038, respectively), were also found. These data further support the immunosuppressive effect of post-RFA serum.

To further locate the key component with immunosuppression effect, and find out whether the immunosuppression effect of post-RFA serum is caused by the components released by necrotic tumor tissue, we removed serum components bigger than 10KD, mostly of which are proteins, in the before-RFA and post-RFA serum with protein chromatography, and treated the PBMC from the same donor for 48h, followed by mass cytometry assay. We detected no population alteration in the post-RFA serum treated PBMC (**Figure 5B**). Meanwhile, we found no alteration in VEGF or CD161 expression, and the expression of MHCII and TLR4 of monocytes showed no difference (**Figure 5D**).

## Discussion

As a method for a precise elimination of tumor loci with minimal invasion and fast recovery, RFA is widely used in the treatment of HCC. Apart from its ability to induce coagulate necrosis directly, RFA also exerts an impact on the anti-tumor immune response (8). The immune phenotype of PBMC, which is the direct reflection of the global immune status, was reported to remarkably correlate with cancer recurrence (3, 9). In this article, we studied the influence of RFA on anti-tumor immune response in the PBMC of recurrent HCC patients using a combination of mass cytometry and high-throughput RNA assay.

### Enhanced antigen-presenting capacity of monocyte after RFA

RFA could activate the antigen-presenting cells by increasing exposure to tumor antigens (10). Following RFA treatment, necrotic HCC cells release tumor specific antigens and other heat stressed cells release acute phase proteins, pro-inflammatory cytokines and heat shock proteins (HSPs) (11, 12). HSPs function as intracellular molecular chaperones facilitating the binding of tumor peptide antigens. The HSP-tumor antigen complex can be effectively recognized by APCs, promoting their maturation (12), In our study, we found that the number of monocytes in peripheral blood of recurrent HCC patients significantly increased after the RFA, and culturing PBMC with post-RFA serum didn’t directly increase the population of monocytes, these findings suggest that RFA could enhance the recruitment of monocytes from the lymphatic organs. Additionally, we discovered that post-RFA serum of recurrent HCC patients induced increased expression of MHCII and TLR4 in monocytes. However, after removing components bigger than 10KD from the post-RFA serum, it demonstrated no influence on the phenotype of monocytes. MHC II and TLR4 are constitutionally expressed by antigen presenting cells and present antigenic peptides to T lymphocytes. MHC II and TLR4 expression correlated significantly with the activity of intratumoral inflammation and the infiltration of CD4+ T lymphocytes (13, 14). These findings suggest that cancer-derived components in the serum of recurrent HCC patients can also enhance the antigen-presenting capacity of monocytes.

### Alteration in peripheral T cell subpopulation and recruitment of T cells

CD4+T cells and CD8+ T cells play pivotal roles in the anti-cancer immune response. Upon recognition of tumor specific antigen presented by APCs, CD4 and CD8 naïve T cells undergo differentiation and become effector T cells. CD4 Teff could induce humeral immune response and enhance cellular immune response by secreting pro-inflammatory cytokines, and CD8 Teff could directly eliminate cancer cells by secreting granzymes, perforin and granulysin. T cell exhaustion has been thought to be an important cause of the failure of anti-cancer immunity. Chronic infection and cancer are believed to be common factors inducing T cell exhaustion (15–17).

An increase in peripheral T cells following RFA has been reported in several studies (18–20), and the increased level of peripheral T cells, especially the number of CD8 T cells, could improve the prognosis of the HCC patients (21). However, our current study found a temporary decrease in peripheral CD4 and CD8 T cells after RFA in recurrent HCC patients, which further declined to lower levels compared to pre-RFA PBMCs. Previous studies suggested that incomplete RFA induces inflammation and accelerates tumor progression (22). In this context, HCC recurrence after RFA, similar to incomplete radiofrequency ablation, may affect inflammation.

We then examined the expression of co-stimulatory molecules in peripheral T cells and found that only a small proportion of CD8 Tnaive showed increased CD28 expression after RFA, and only one out of four CD4 Tnaive clusters showed increased ICOS expression. Also, no alterations in OX40 and 4-IBB expression were found. Meanwhile, we found that the post-RFA serum of recurrent HCC patients could reduce population of CD8 Tem and CD8 Teff, and the CD161 expression in CD4 Teff, CD4 Tnaive, CD8 Tem, CD8Teff, and CD8 Tnaive. CD161 is a marker of all human IL17 producing T cell subsets, and it is also a pan-cancer prognostic factor with favoring outcome (23, 24), and these inhibitory function of RFA over T cells could be eliminated by removal of serum components which is bigger than 10KD. We also found the down-regulation of mRNA expression of GZMB (granzyme B), GZMK (granzyme K), GZMH (granzyme H), which are key enzymes in the cytotoxic function of CD8+T cells (25, 26), and the down-regulation of ISG15 (Interferon-Stimulated Gene 15), NKG7 (natural killer cell granule protein 7), which were reported to be related to T cell and NK cell related immune response (27). RFA was reported to enhance host immune response by increasing tumor-associated antigen (TAA)-specific T cell responses (3, 28). But on the contrary, we observed a significant reduction in the population and cytotoxic function of CD8 Teff and CD8 Tem cells after incomplete RFA, despite these cells may receive more tumor antigens because of the enhancement of the antigen-presenting capacity of monocytes in incomplete RFA. These findings suggest that the inhibitory effect of proteins secreted by necrotic tumor tissues may be an important reason for inhibition of T cell- related anti-tumor response and tumor recurrence. In recent years targeting immune checkpoint therapy showed promising therapeutic effect in HCC. Wen et al. (29) reported that combining RFA with anti-PD-1 showed improved RFS and was deemed safe for patients with recurrent HCC who had previously undergone RFA treatment alone. But some studies also suggest that incomplete radiofrequency ablation accelerates tumor progression and hinders PD-1 immunotherapy by inducing the accumulation of tumor-associated macrophages. Overall, the combination of RFA and anti-PD-1 therapy represents a promising treatment strategy for recurrent HCC patients, but further studies are warranted to enhance the efficacy of this treatment approach.

### Up-regulation of VEGF in post-RFA serum treated lymphocytes

In this study, we observed a significant increase in VEGF production in monocytes, Tregs, B cells and CD4 Tnaive cells showed significant increase following treated by post-RFA serum, and this manifest could also be eliminated by removal of serum components which is bigger than 10KD. VEGF is best known for its role in the tumor angiogenesis, and it is known to be extensively secreted by tumor associated macrophages and Tregs (30). It has also been reported to selectively kill CD8+T cells and produce a immunosuppressant microenvironment for cancer cells (31). These findings suggest that cancer-derived components in the serum may contribute to enhanced immunosuppression and angiogenesis by promoting the secretion of VEGF.

In conclusion, by using a combination of mass cytometry and high-throughput mRNA assay, our study demonstrated that in recurrent HCC patients, RFA therapy can enhance the antigen-presenting capacity of monocytes, However, it is insufficient to induce a robust anti-tumor immune response. This is because RFA therapy can inhibit the anti-tumor immune response by declining the population of T cells, inhibiting the activation of T cells and downregulating the expression of CD161 and CD5 in various T cell subpopulations. Additionally, tumor-derived components contribute to the creation of an immunosuppressive microenvironment by promoting the secretion of VEGF in Monocytes, Tregs, B cells and CD4 T naïve cells. Therefore, the immunosuppression caused by tumor-derived big molecules may be an important reason of HCC recurrence after RFA therapy, and application of immunopotentiating therapy in recurrent HCC patients should be further studied.

## Data availability statement

The raw data supporting the conclusions of this article will be made available by the authors, without undue reservation.

## Ethics statement

The studies involving humans were approved by Medical Ethics Committee of YouAn Hospital. The studies were conducted in accordance with the local legislation and institutional requirements. The participants provided their written informed consent to participate in this study.

## Author contributions

YZ: Data curation, Formal Analysis, Writing – original draft, Writing – review & editing. TY: Methodology, Investigation, Writing – original draft, Writing – review & editing. YO: Methodology, Investigation, Writing – original draft, Writing – review & editing. FW: Methodology, Investigation, Writing – original draft, Writing – review & editing. DL: Methodology, Investigation, Writing – original draft, Writing – review & editing. LQ: Methodology, Investigation, Writing – original draft, Writing – review & editing. ZX: Methodology, Investigation, Writing – original draft, Writing – review & editing. WR: Resources, Writing – original draft, Writing – review & editing. KL: Resources, Writing – original draft, Writing – review & editing. JZ: Data curation, Writing – original draft, Writing – review & editing. FL: Data curation, Writing – original draft, Writing – review & editing. YS: Data curation, Writing – original draft, Writing – review & editing. ZY: Formal Analysis, Methodology, Project administration, Supervision, Writing – review & editing. DC: Conceptualization, Funding acquisition, Methodology, Project administration, Resources, Supervision, Writing – review & editing. WW: Conceptualization, Data curation, Investigation, Methodology, Supervision, Writing – review & editing.

## Supplementary material

Supplementary Figure 1 Significant altered subpopulation of PBMC identified by manual gating **(A)** Population of B cells significantly reduced after the first RFA (P=0.021), and the B cell count remained at a low level before and after the secondary RFA. **(B)** The percentage of monocytes showed a fast increase after RFA, and was reduced to the normal level before the second RFA (P=0.026). **(C)** and **(D)**. The percentage of CD4+ T cells and CD8 Tcells in CD45+ cells was significantly reduced after both the first and the second RFA (P=0.024 and 0.03, respectively).

Supplementary Figure 2 Expression of clustering markers of SPADE nodes in patients one. Median marker expression were marked using the color of the nodes and cell counts were marked with the cell count of the nodes. The cell type of every nodes were identified according to its clustering marker expression. The expression of nodes with small cell counts may vary slightly and these nodes are annotated according to the expression of these nodes in other fcs files with larger cell count.

Supplementary Figure 3 Clustering markers and functional markers of T cells. A viSNE analysis was performed on CD3+ T cells. 10 T cell related cells markers were included in the analysis to generate two variables (tSNE1 and tSNE2) to differentiate the T cells populations. Plots are colored according to 10 populational and functional markers. Also, CXCR3 and CCR4+ showed similar expression pattern in all these five patients.

Supplementary Figure 4 CTLA4 and CD28 expression of T cells of patients 9. Compared with other patients, patient 9 showed remarkable CTLA-4 expression in CD3+ T cells and the expression of CTLA-4 greatly reduced, accompanied with increase of CD28 expression in time point 3 and 4, while in the other four patients, the CTLA-4 were mainly expressed in DPT cells.
