# Supplementary figures and images for: Radiofrequency ablation plays double role in immunosuppression and activation of PBMCs in recurrent hepatocellular carcinoma
Source: Front Immunol. 2024 Jan 29;15:1339213. doi: 10.3389/fimmu.2024.1339213 (PMC10859425; doi:10.3389/fimmu.2024.1339213)

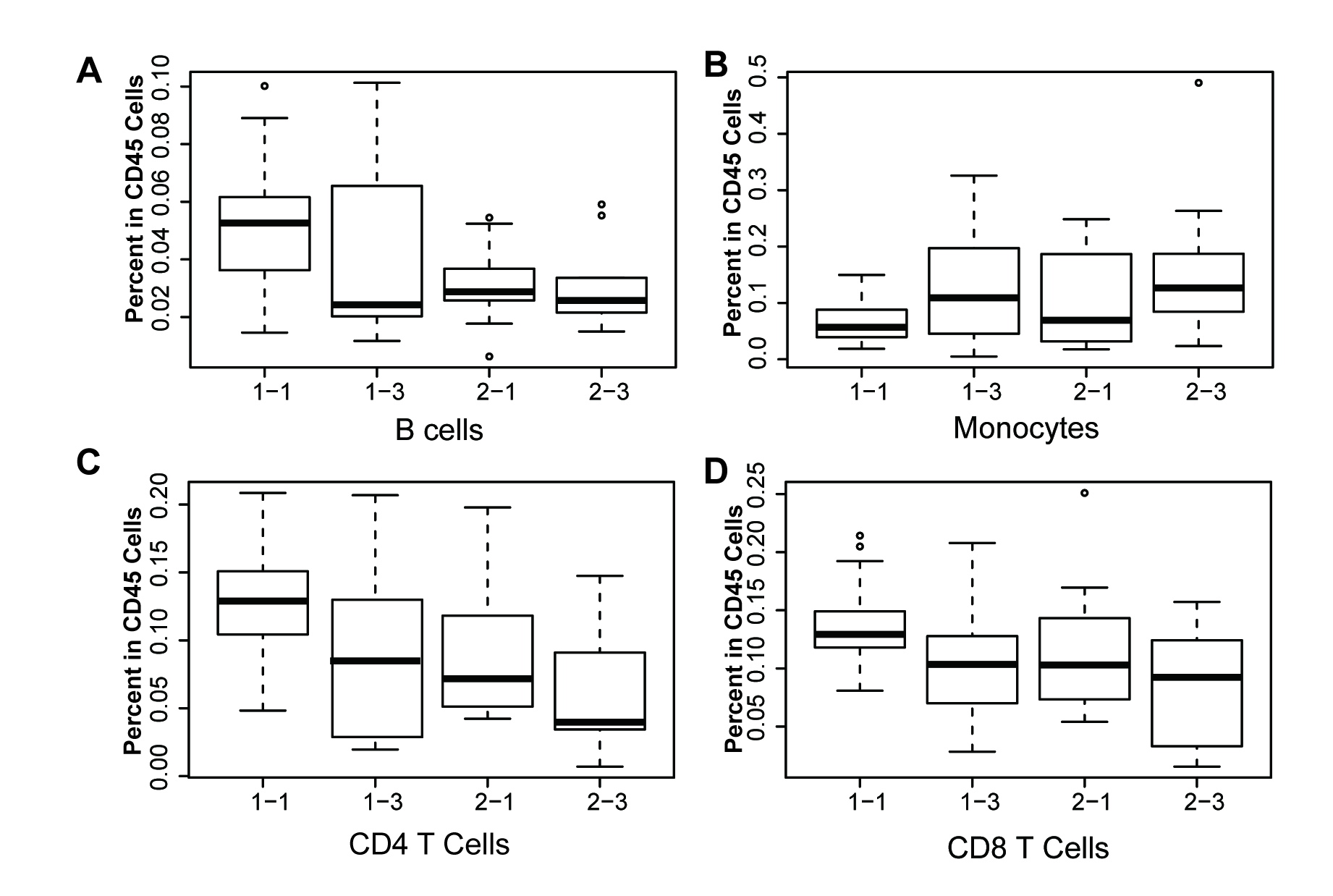

Supplement: Supplementary Figure 1 — Significant altered subpopulation of PBMC identified by manual gating (A) Population of B cells significantly reduced after the first RFA (P=0.021), and the B cell count remained at a low level before and after the secondary RFA. (B) The percentage of monocytes showed a fast increase after RFA, and was reduced to the normal level before the second RFA (P=0.026). (C) and (D). The percentage of CD4+ T cells and CD8 Tcells in CD45+ cells was significantly reduced after both the first and the second RFA (P=0.024 and 0.03, respectively). [file Image_1.tif]

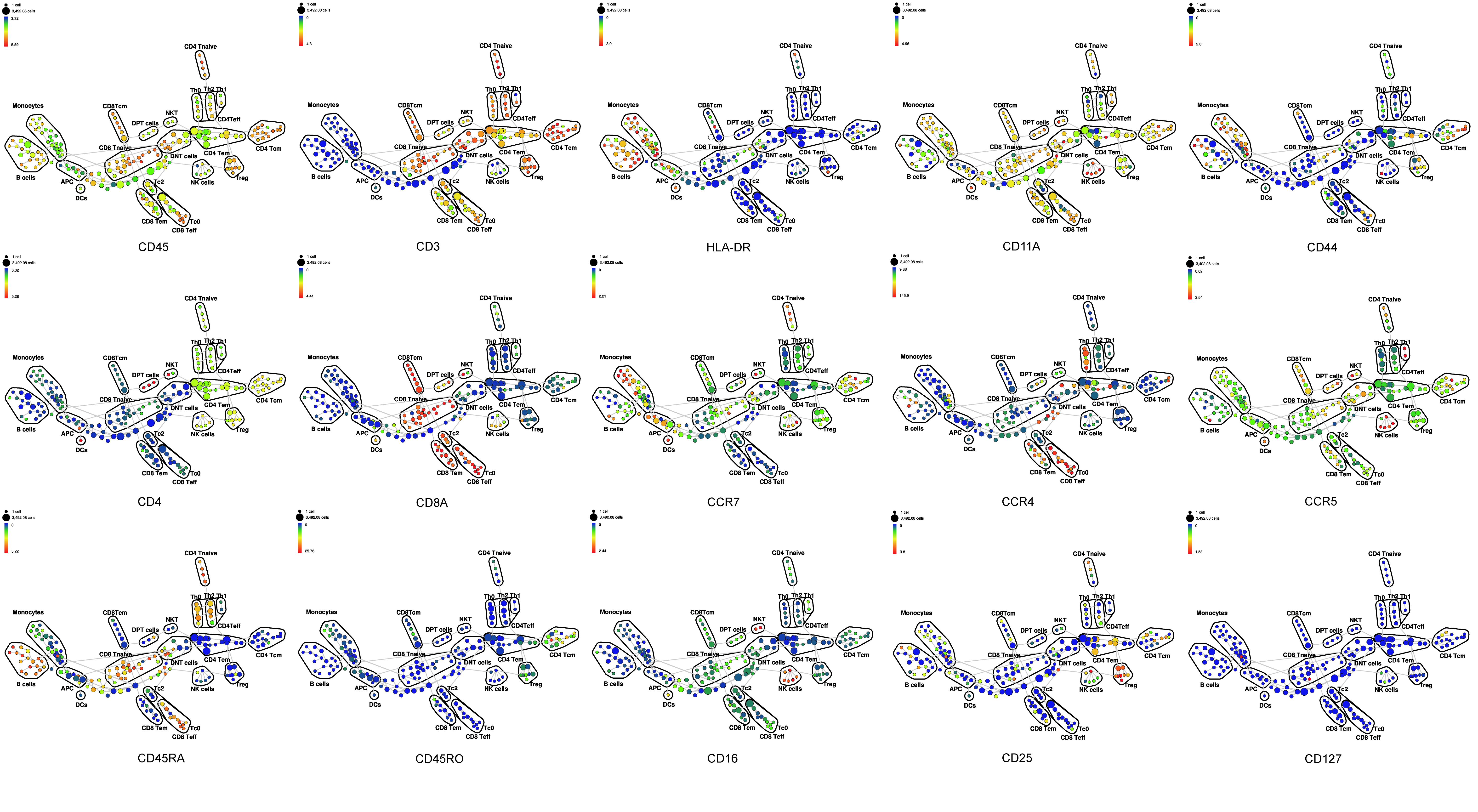

Supplement: Supplementary Figure 2 — Expression of clustering markers of SPADE nodes in patients one. Median marker expression were marked using the color of the nodes and cell counts were marked with the cell count of the nodes. The cell type of every nodes were identified according to its clustering marker expression. The expression of nodes with small cell counts may vary slightly and these nodes are annotated according to the expression of these nodes in other fcs files with larger cell count. [file Image_2.tif]

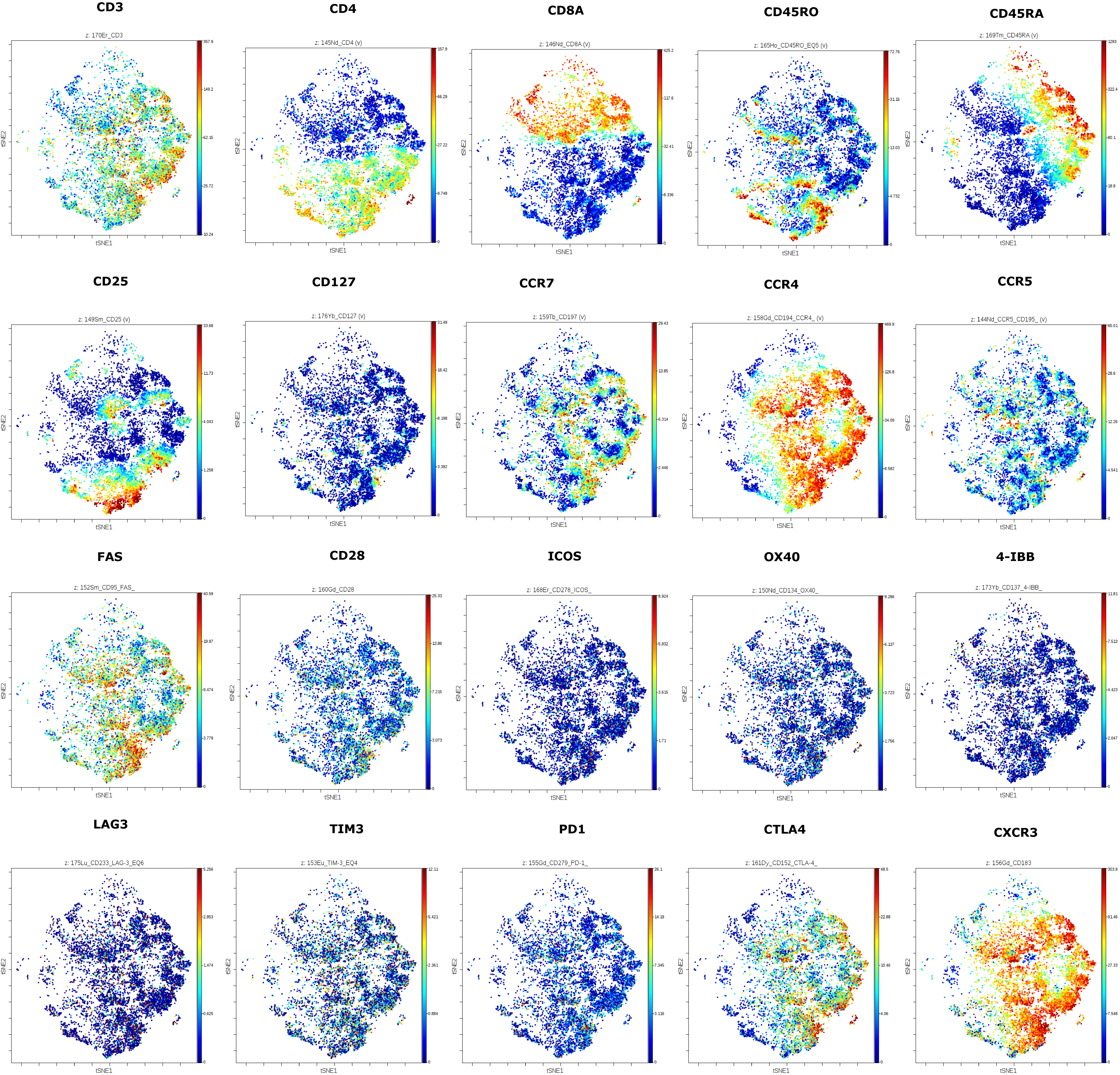

Supplement: Supplementary Figure 3 — Clustering markers and functional markers of T cells. A viSNE analysis was performed on CD3+ T cells. 10 T cell related cells markers were included in the analysis to generate two variables (tSNE1 and tSNE2) to differentiate the T cells populations. Plots are colored according to 10 populational and functional markers. Also, CXCR3 and CCR4+ showed similar expression pattern in all these five patients. [file Image_3.tif]

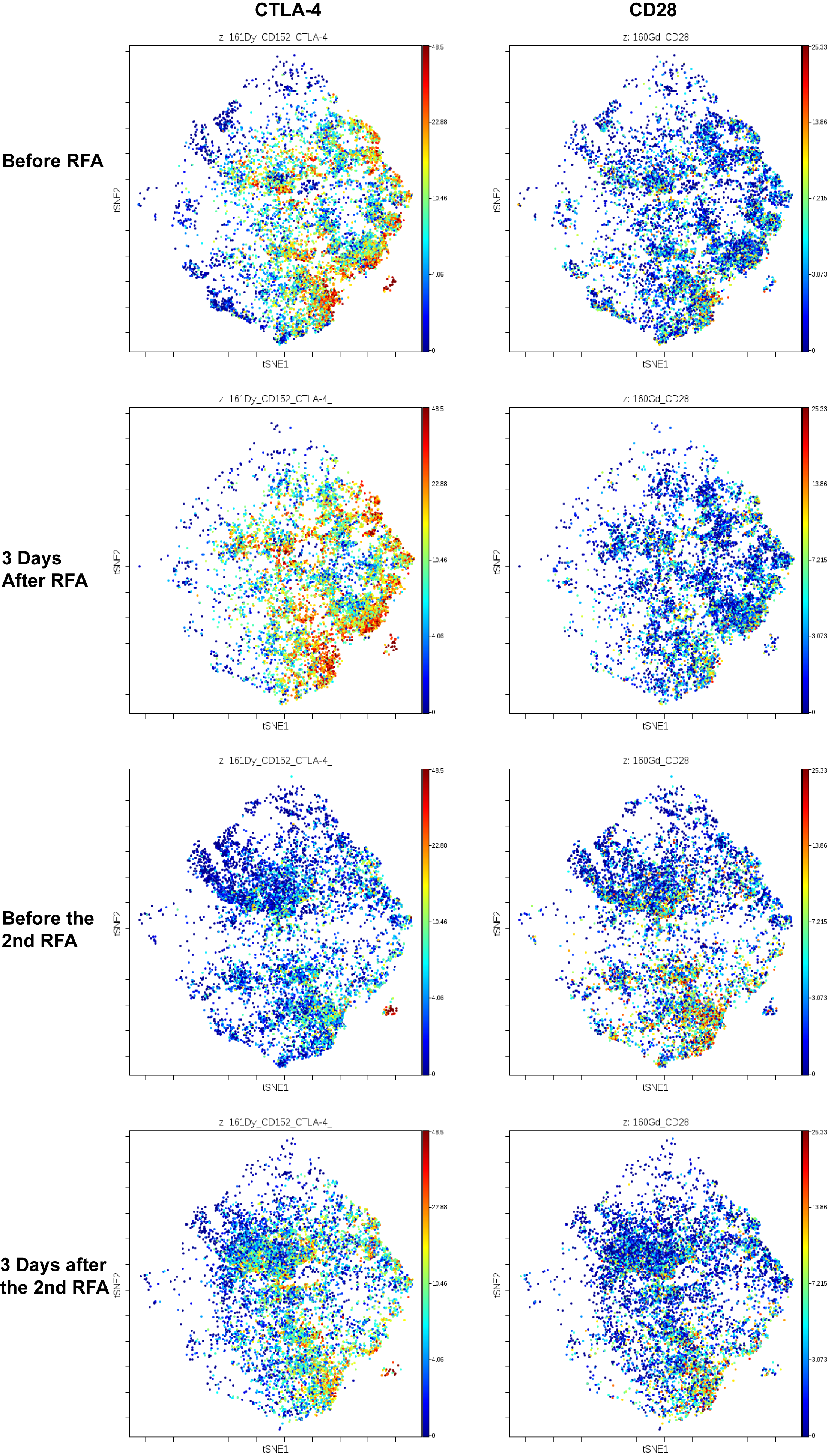

Supplement: Supplementary Figure 4 — CTLA4 and CD28 expression of T cells of patients 9. Compared with other patients, patient 9 showed remarkable CTLA-4 expression in CD3+ T cells and the expression of CTLA-4 greatly reduced, accompanied with increase of CD28 expression in time point 3 and 4, while in the other four patients, the CTLA-4 were mainly expressed in DPT cells. [file Image_4.tif]
