# Supplementary material for: Radiofrequency ablation plays double role in immunosuppression and activation of PBMCs in recurrent hepatocellular carcinoma
Source: Front Immunol. 2024 Jan 29;15:1339213. doi: 10.3389/fimmu.2024.1339213 (PMC10859425; doi:10.3389/fimmu.2024.1339213)
Supplement: Supplementary file 5 [file DataSheet_1.doc]

###This code could used in the repeated measurement ANOVA analysis of cyTof data.

library(car)

###create empty result dataframe

SUM=data.frame(P=c(NA),mauchly.p=c(NA),GG.p=c(NA),HF.p=c(NA),stringsAsFactors = F)

SPADEresult<-data.frame(stringsAsFactors = F)

meansd<-data.frame(cbind("time1","time2","time3","time4"), stringsAsFactors = F)

colnames(meansd)<-meansd[1,]

meansd<-meansd[-1,]

#read and transform data

rawdata<-read.csv("4IBB.csv",header = T,stringsAsFactors = F)

rawdata<-rawdata[,c(1,2,3,4,5,6,7,8,9,10,11,12,13,18,19,20,21,22,23,24,25,26,27,28,29,34,35,36,37,42,43,44,45,46,47,48,49,50,51,52,53,54,55,56,57,58,59,60,61,62,63,64,65)]

trawdata<-data.frame(t(rawdata))

colnames(trawdata)<-trawdata[1,]

trawdata<-trawdata[-1,]

trawdata$time<-rep(1:4,13)

trawdata$id<-c(1,1,1,1,2,2,2,2,3,3,3,3,4,4,4,4,5,5,5,5,6,6,6,6,7,7,7,7,8,8,8,8,9,9,9,9,10,10,10,10,11,11,11,11,12,12,12,12,13,13,13,13)

group <- factor(c("g1","g2","g3","g4"))

#calculate

for(i in 1:200) {testdata<-cbind(trawdata[,i][trawdata$time==1],trawdata[,i][trawdata$time==2],trawdata[,i][trawdata$time==3],trawdata[,i][trawdata$time==4])

meansd[i,]<-cbind(mean(trawdata[,i][trawdata$time==1],na.rm = T),mean(trawdata[,i][trawdata$time==2],na.rm = T),mean(trawdata[,i][trawdata$time==3],na.rm = T),mean(trawdata[,i][trawdata$time==4],na.rm = T))

modelx <- lm(testdata ~ 1)

mlm1.aov <- Anova(modelx, idata = data.frame(group),idesign = ~group, type="III")

noderesult<-summary(mlm1.aov, multivariate=FALSE)

SUM[i,]<-c(noderesult$univariate.tests[2,6],noderesult$sphericity.tests[2],noderesult$pval.adjustments[1,2],noderesult$pval.adjustments[1,4])

}

#write results to file

CD9<-ifelse(SUM$mauchly.p<0.05,SUM$GG.p,SUM$P)

SPADEresult<-data.frame(cbind(percent,count,IBB4,CD49D,CD57,CD69,CD127,CD161,CD183,CTLA4,PD1,TIM3,FAS,ICOS,LAG3,OX40,CD2,CD5,CD7,CD9,CCR4,CCR5,CCR7,HLADR,CD3,CD4,CD8A,CD11A,CD16,CD25,CD27,CD28,CD44,CD45RA,CD45RO))

result.CTLA4<-meansd

#save results as csv files

write.csv(SPADEresult,"SPADEresult2.csv")

write.csv(detailresult,"SPADEresult2.csv")
