# Supplementary material for: Radiofrequency ablation plays double role in immunosuppression and activation of PBMCs in recurrent hepatocellular carcinoma
Source: Front Immunol. 2024 Jan 29;15:1339213. doi: 10.3389/fimmu.2024.1339213 (PMC10859425; doi:10.3389/fimmu.2024.1339213)
Supplement: Supplementary file 6 [file DataSheet_2.docx]

| Node | Cell Type | P value | Time Point1 | Time Point2 | Time Point3 | Time Point4 |
| --- | --- | --- | --- | --- | --- | --- |
| 1 | CD8Tnaive | 0.267 | 157(61-263) | 57(24-257) | 125(32-166) | 70(22-170) |
| 2 | Tc2 | 0.19 | 814(359-1660) | 356(155-865) | 301(185-922) | 478(87-922) |
| 3 | Monocytes | 0.237 | 661(93-1301) | 96(52-688) | 165(12-634) | 137(45-478) |
| 4 | B cells | 0.017* | 552(237-1714) | 178(50-290) | 253(135-435) | 101(49-200) |
| 5 | CD8Tnaive | 0.014* | 1475(630-2987) | 453(146-850) | 562(239-1188) | 310(68-714) |
| 6 | CD4Tem | 0.035* | 1175(609-2770) | 306(149-539) | 522(284-986) | 513(96-966) |
| 7 | UC | 0.267 | 385(111-1079) | 158(54-594) | 145(24-554) | 111(44-401) |
| 8 | CD8Tnaive | 0.073 | 662(512-1203) | 319(78-444) | 263(111-639) | 269(131-441) |
| 9 | UC | 0.235 | 195(85-519) | 84(58-310) | 75(54-471) | 87(48-188) |
| 10 | CD8Tnaive | 0.021* | 440(314-578) | 151(91-250) | 119(88-353) | 129(42-366) |
| 11 | Monocytes | 0.658 | 734(288-1015) | 840(347-1115) | 549(188-933) | 945(253-1294) |
| 12 | Monocytes | 0.668 | 101(48-191) | 26(7-102) | 47(21-190) | 42(32-209) |
| 13 | CD8Tem | 0.103 | 641(205-1926) | 129(29-664) | 372(113-569) | 216(30-441) |
| 14 | CD8Tnaive | 0.030* | 1188(314-1460) | 274(114-646) | 212(108-458) | 176(62-964) |
| 15 | CD8Tem | 0.089 | 263(51-942) | 34(10-162) | 82(26-237) | 66(10-159) |
| 16 | UC | 0.175 | 354(69-903) | 34(15-730) | 72(14-1064) | 110(17-386) |
| 17 | Monocytes | 0.315 | 270(74-764) | 120(44-292) | 190(34-266) | 133(40-353) |
| 18 | CD4Tem | 0.055 | 722(221-1312) | 110(55-515) | 118(99-753) | 221(59-383) |
| 19 | CD4Tcm | 0.092 | 113(79-220) | 44(16-118) | 76(66-184) | 78(26-125) |
| 20 | DNT | 0.252 | 205(135-722) | 77(36-256) | 66(35-96) | 83(8-341) |
| 21 | CD8Tcm | 0.56 | 189(123-448) | 128(50-405) | 110(81-250) | 140(44-526) |
| 22 | CD8Tnaive | 0.056 | 1158(603-1525) | 394(279-754) | 384(132-792) | 290(64-488) |
| 23 | CD4Tem | 0.011* | 3261(1327-5023) | 661(427-1413) | 799(543-2224) | 464(186-1341) |
| 24 | Monocytes | 0.753 | 276(119-323) | 165(126-529) | 208(42-568) | 366(102-433) |
| 25 | CD4Tem | 0.013* | 994(375-1729) | 167(61-593) | 270(148-933) | 306(134-510) |
| 26 | Tc0 | 0.022* | 697(260-2861) | 112(27-905) | 339(81-848) | 110(34-632) |
| 27 | CD8Tcm | 0.372 | 100(81-360) | 45(21-98) | 47(22-317) | 117(19-204) |
| 28 | Monocytes | 0.601 | 400(120-652) | 191(116-331) | 457(155-531) | 394(230-728) |
| 29 | APC | 0.171 | 90(26-100) | 32(19-66) | 44(33-61) | 29(22-65) |
| 30 | CD8Tem | 0.159 | 324(132-1332) | 64(30-258) | 142(71-298) | 101(23-246) |
| 31 | Monocytes | 0.788 | 448(268-1597) | 417(281-817) | 470(149-2420) | 572(262-1557) |
| 32 | Th2 | 0.098 | 846(123-1365) | 127(28-358) | 180(47-453) | 120(51-205) |
| 33 | CD4Tem | 0.036* | 641(372-2373) | 577(100-815) | 323(233-884) | 323(101-609) |
| 34 | UC | 0.415 | 116(40-218) | 101(36-119) | 41(29-191) | 63(34-140) |
| 35 | UC | 0.024* | 1527(263-2230) | 131(65-966) | 266(50-549) | 135(35-645) |
| 36 | UC | 0.135 | 1742(900-2544) | 504(344-1837) | 981(364-1671) | 721(135-2329) |
| 37 | Tc0 | 0.323 | 278(234-841) | 61(14-166) | 135(16-230) | 33(5-108) |
| 38 | Th2 | 0.017* | 1291(552-1384) | 188(152-386) | 276(214-967) | 310(79-445) |
| 39 | CD4Tcm | 0.448 | 154(68-318) | 59(15-90) | 81(66-186) | 25(20-153) |
| 40 | Tc0 | 0.513 | 137(39-304) | 26(5-145) | 39(23-104) | 45(11-91) |
| 41 | B cells | 0.020* | 501(120-1339) | 28(7-297) | 133(18-381) | 82(21-124) |
| 42 | DNT | 0.205 | 602(169-952) | 97(64-532) | 152(69-481) | 92(80-351) |
| 43 | CD8Tcm | 0.029* | 652(328-985) | 171(81-708) | 224(149-448) | 251(68-727) |
| 44 | UC | 0.571 | 752(189-1465) | 408(205-1785) | 646(141-1477) | 630(207-3446) |
| 45 | Tc0 | 0.116 | 774(283-1565) | 84(24-434) | 216(25-474) | 67(42-170) |
| 46 | CD4Tem | 0.082 | 1391(327-2149) | 253(46-1692) | 467(171-1357) | 123(65-349) |
| 47 | B cells | 0.068 | 249(119-305) | 71(35-101) | 131(83-248) | 90(32-252) |
| 48 | Monocytes | 0.487 | 363(98-1725) | 102(26-433) | 125(26-1028) | 177(22-433) |
| 49 | CD4Tem | 0.27 | 419(308-1334) | 301(57-638) | 247(78-357) | 197(33-332) |
| 50 | Th0 | 0.08 | 286(148-708) | 61(16-500) | 76(17-420) | 53(17-103) |
| 51 | APC | 0.371 | 50(36-91) | 27(15-62) | 21(16-69) | 42(22-48) |
| 52 | CD4Tem | 0.002** | 1295(394-1591) | 175(59-662) | 468(286-776) | 207(76-411) |
| 53 | CD4Tcm | 0.002** | 866(441-2078) | 231(55-647) | 291(248-670) | 223(99-392) |
| 54 | UC | 0.157 | 622(299-4060) | 745(145-1473) | 233(151-3205) | 341(192-832) |
| 55 | Monocytes | 0.343 | 442(81-614) | 120(73-203) | 295(115-390) | 159(101-540) |
| 56 | CD8Tem | 0.407 | 288(67-414) | 66(13-92) | 155(57-267) | 103(16-224) |
| 57 | CD8Tnaive | 0.037* | 353(218-847) | 115(33-309) | 131(83-516) | 111(24-277) |
| 58 | Th2 | 0.026* | 1833(340-3001) | 287(180-720) | 425(127-1371) | 240(113-466) |
| 59 | DNT | 0.211 | 190(66-813) | 59(15-566) | 88(24-593) | 55(15-317) |
| 60 | B cells | 0.165 | 647(155-914) | 25(11-306) | 205(26-293) | 81(42-325) |
| 61 | CD8Tnaive | 0.023* | 844(203-2081) | 247(68-1202) | 298(103-1052) | 325(121-584) |
| 62 | NKs | 0.56 | 3(2-15) | 7(3-11) | 5(1-7) | 4(2-8) |
| 63 | CD4Tcm | 0.276 | 20(14-32) | 3(1-24) | 6(4-29) | 3(3-11) |
| 64 | Monocytes | 0.802 | 131(29-695) | 64(22-176) | 91(30-457) | 129(44-388) |
| 65 | UC | 0.067 | 512(301-1087) | 210(122-318) | 204(73-1028) | 201(65-365) |
| 66 | Monocytes | 0.425 | 716(260-1274) | 491(253-667) | 797(332-1006) | 437(273-1238) |
| 67 | CD8Tcm | 0.127 | 219(59-343) | 51(20-333) | 74(55-170) | 64(40-177) |
| 68 | CD4Tcm | 0.173 | 220(153-346) | 39(25-250) | 127(72-691) | 105(28-360) |
| 69 | APC | 0.217 | 255(161-701) | 163(51-283) | 171(122-388) | 147(57-350) |
| 70 | Th0 | 0.21 | 319(93-384) | 50(15-153) | 82(28-222) | 49(13-165) |
| 71 | Tc2 | 0.49 | 288(136-535) | 162(29-317) | 102(60-140) | 131(31-312) |
| 72 | UC | 0.102 | 936(430-2869) | 363(226-1011) | 406(92-2483) | 243(161-803) |
| 73 | Th2 | 0.025* | 1348(339-2121) | 208(56-635) | 236(116-781) | 221(30-637) |
| 74 | Monocytes | 0.328 | 155(50-267) | 82(28-142) | 109(47-155) | 103(37-129) |
| 75 | Tc0 | 0.236 | 439(370-1150) | 107(21-344) | 154(47-410) | 42(23-246) |
| 76 | DCs | 0.431 | 62(20-91) | 40(12-70) | 80(47-122) | 54(31-98) |
| 77 | Treg | 0.057 | 225(143-901) | 135(31-195) | 114(62-473) | 191(73-262) |
| 78 | DNT | 0.004** | 937(333-1974) | 262(97-1167) | 293(108-619) | 189(56-769) |
| 79 | UC | 0.763 | 792(228-1622) | 651(230-1232) | 656(118-947) | 762(233-2612) |
| 80 | CD4Tcm | 0.209 | 184(137-961) | 54(25-250) | 104(78-195) | 57(20-128) |
| 81 | CD8Tnaive | 0.1 | 918(208-1385) | 230(69-1400) | 372(150-1010) | 254(131-414) |
| 82 | B cells | 0.343 | 40(20-43) | 4(1-24) | 14(4-50) | 5(1-107) |
| 83 | Tc0 | 0.015* | 809(477-2116) | 215(29-891) | 242(58-1014) | 94(30-323) |
| 84 | Monocytes | 0.214 | 403(356-738) | 263(120-483) | 313(162-670) | 305(129-645) |
| 85 | B cells | 0.022* | 1463(472-3492) | 226(63-1405) | 728(224-1352) | 277(196-672) |
| 86 | Monocytes | 0.761 | 740(235-974) | 465(246-611) | 711(229-1338) | 968(205-1580) |
| 87 | UC | 0.138 | 38(16-72) | 47(8-78) | 57(6-132) | 59(16-125) |
| 88 | B cells | 0.019* | 479(284-1303) | 161(59-246) | 302(141-346) | 118(79-171) |
| 89 | Monocytes | 0.272 | 172(73-266) | 80(35-194) | 152(83-310) | 107(64-263) |
| 90 | Monocytes | 0.479 | 230(134-761) | 283(231-861) | 341(65-437) | 418(178-573) |
| 91 | Monocytes | 0.63 | 264(79-545) | 146(85-310) | 260(97-583) | 352(76-503) |
| 92 | CD8Tnaive | 0.004** | 1748(1013-3284) | 743(137-1517) | 666(227-2091) | 391(107-932) |
| 93 | CD8Tnaive | 0.014* | 515(455-1032) | 240(53-432) | 237(74-612) | 196(67-268) |
| 94 | UC | 0.713 | 332(131-439) | 117(45-309) | 211(119-366) | 163(97-412) |
| 95 | CD4Tem | 0.085 | 1021(367-2279) | 149(40-743) | 156(113-1281) | 134(34-447) |
| 96 | UC | 0.794 | 169(63-481) | 192(34-405) | 157(34-446) | 151(58-490) |
| 97 | CD4Tem | 0.275 | 180(75-473) | 53(18-183) | 63(26-288) | 69(15-97) |
| 98 | NKs | 0.445 | 55(34-90) | 28(23-74) | 55(25-105) | 35(19-108) |
| 99 | APC | 0.408 | 243(180-355) | 271(84-921) | 286(38-408) | 506(93-1037) |
| 100 | DNT | 0.118 | 55(26-156) | 35(11-50) | 55(21-87) | 26(11-101) |
| 101 | CD8Tnaive | 0.042* | 463(365-1272) | 135(40-263) | 183(69-282) | 165(16-326) |
| 102 | Treg | 0.103 | 319(188-549) | 51(28-145) | 78(53-185) | 109(36-152) |
| 103 | DNT | 0.146 | 216(117-764) | 62(22-406) | 153(45-788) | 84(28-134) |
| 104 | Tc0 | 0.175 | 305(175-593) | 64(18-187) | 131(21-229) | 45(19-141) |
| 105 | UC | 0.345 | 266(145-640) | 227(92-831) | 181(35-282) | 377(99-1777) |
| 106 | B cells | 0.023* | 243(144-636) | 69(25-133) | 105(20-208) | 55(39-131) |
| 107 | B cells | 0.023* | 117(30-288) | 10(1-22) | 27(1-53) | 17(11-23) |
| 108 | UC | 0.825 | 127(65-241) | 124(39-454) | 94(16-197) | 166(26-710) |
| 109 | CD4Tem | 0.147 | 445(344-1338) | 139(108-222) | 150(87-375) | 140(48-397) |
| 110 | Monocytes | 0.19 | 218(103-373) | 75(49-135) | 170(105-227) | 150(50-309) |
| 111 | B cells | 0.036* | 356(113-1100) | 111(19-237) | 106(67-262) | 64(36-114) |
| 112 | CD4Tnaive | 0.023* | 74(30-170) | 10(9-51) | 43(24-57) | 19(11-41) |
| 113 | UC | 0.213 | 395(62-1207) | 29(6-273) | 113(16-1012) | 84(14-304) |
| 114 | CD8Tnaive | 0.492 | 62(22-94) | 14(7-31) | 29(14-50) | 22(7-30) |
| 115 | Th0 | 0.059 | 633(200-1938) | 66(43-577) | 188(67-669) | 94(37-193) |
| 116 | Monocytes | 0.725 | 274(106-428) | 237(159-699) | 247(161-699) | 298(115-707) |
| 117 | CD4Tcm | 0.063 | 819(375-1196) | 88(66-360) | 259(135-726) | 202(77-415) |
| 118 | CD4Tcm | 0.151 | 163(122-776) | 33(11-205) | 168(62-373) | 104(34-150) |
| 119 | UC | 0.368 | 83(40-473) | 95(9-366) | 64(13-103) | 44(18-248) |
| 120 | UC | 0.265 | 138(102-783) | 107(17-408) | 120(31-348) | 91(15-213) |
| 121 | UC | 0.255 | 246(152-879) | 155(57-225) | 94(36-299) | 114(21-304) |
| 122 | Tc0 | 0.557 | 28(6-145) | 4(1-33) | 25(8-44) | 10(8-29) |
| 123 | Monocytes | 0.99 | 250(69-438) | 177(69-340) | 260(100-485) | 215(131-405) |
| 124 | APC | 0.912 | 662(201-968) | 416(287-948) | 394(151-799) | 470(256-1272) |
| 125 | B cells | 0.039* | 391(178-742) | 72(14-151) | 153(138-236) | 72(39-123) |
| 126 | CD4Tcm | 0.3 | 301(155-619) | 53(19-184) | 156(83-372) | 80(25-391) |
| 127 | NKT | 0.387 | 99(91-388) | 49(38-182) | 142(53-283) | 98(49-215) |
| 128 | Treg | 0.127 | 84(47-242) | 45(23-85) | 68(30-152) | 36(24-79) |
| 129 | B cells | 0.016* | 602(83-1166) | 26(7-600) | 162(21-269) | 114(25-149) |
| 130 | NKs | 0.541 | 19(1-48) | 6(0-49) | 12(0-25) | 17(3-31) |
| 131 | CD4Tnaive | 0.159 | 334(165-779) | 49(21-189) | 112(57-319) | 137(23-233) |
| 132 | Tc0 | 0.006** | 223(45-1027) | 49(11-321) | 66(24-334) | 44(16-142) |
| 133 | CD8Tnaive | 0.458 | 209(81-465) | 93(24-618) | 118(26-246) | 92(33-185) |
| 134 | UC | 0.218 | 752(111-1177) | 264(41-769) | 205(62-549) | 136(23-377) |
| 135 | CD4Tem | 0.05 | 103(45-188) | 42(18-111) | 57(29-88) | 67(44-77) |
| 136 | CD8Tnaive | 0.008** | 1227(359-2528) | 263(92-641) | 368(138-858) | 169(86-380) |
| 137 | B cells | 0.003** | 642(110-1048) | 51(31-139) | 108(39-156) | 59(9-103) |
| 138 | B cells | 0.112 | 247(55-361) | 4(3-71) | 41(10-68) | 17(8-51) |
| 139 | APC | 0.457 | 55(47-150) | 71(45-155) | 34(15-94) | 104(27-167) |
| 140 | DPT | 0.394 | 120(74-222) | 91(27-166) | 118(34-185) | 107(36-200) |
| 141 | Tc2 | 0.277 | 321(128-355) | 111(74-141) | 73(59-230) | 108(33-404) |
| 142 | CD8Tnaive | 0.087 | 34(5-65) | 18(6-32) | 12(5-29) | 10(5-28) |
| 143 | UC | 0.244 | 291(55-589) | 21(11-535) | 137(14-236) | 38(24-225) |
| 144 | Monocytes | 0.614 | 217(65-457) | 170(92-240) | 168(47-225) | 101(76-316) |
| 145 | CD4Tem | 0.116 | 788(310-2584) | 296(139-549) | 320(126-548) | 429(54-1026) |
| 146 | CD4Tem | 0.379 | 118(21-179) | 25(6-246) | 44(29-114) | 40(16-114) |
| 147 | Treg | 0.046* | 863(285-2387) | 279(53-786) | 296(192-955) | 474(126-698) |
| 148 | DNT | 0.039* | 286(138-525) | 96(43-258) | 175(56-212) | 105(70-234) |
| 149 | DNT | 0.2 | 464(227-642) | 93(21-804) | 352(41-718) | 129(54-181) |
| 150 | UC | 0.351 | 36(12-62) | 13(8-81) | 29(5-55) | 20(6-55) |
| 151 | Th0 | 0.287 | 339(45-1015) | 55(8-441) | 60(15-257) | 32(2-105) |
| 152 | CD8Tem | 0.201 | 260(61-503) | 69(7-254) | 108(30-153) | 72(24-193) |
| 153 | CD4Tcm | 0.085 | 378(233-583) | 232(45-327) | 281(132-408) | 167(107-278) |
| 154 | CD8Tem | 0.392 | 278(76-732) | 65(5-490) | 62(35-292) | 106(12-185) |
| 155 | CD8Tnaive | 0.142 | 197(123-398) | 98(36-163) | 108(36-225) | 69(21-165) |
| 156 | APC | 0.771 | 118(52-426) | 68(30-460) | 92(15-424) | 77(38-698) |
| 157 | Monocytes | 0.773 | 79(69-167) | 127(57-266) | 67(40-149) | 150(36-333) |
| 158 | APC | 0.655 | 796(257-1363) | 454(269-1114) | 564(187-1445) | 556(266-1326) |
| 159 | DNT | 0.144 | 305(85-636) | 86(30-228) | 189(57-270) | 65(42-235) |
| 160 | CD4Tcm | 0.021* | 775(366-1277) | 119(70-704) | 312(183-645) | 181(89-397) |
| 161 | B cells | 0.017* | 344(83-702) | 71(20-214) | 110(26-194) | 38(22-93) |
| 162 | CD8Tcm | 0.612 | 100(22-160) | 22(15-156) | 55(14-80) | 29(18-110) |
| 163 | CD8Tem | 0.513 | 556(81-798) | 152(21-710) | 153(53-316) | 130(26-526) |
| 164 | Tc0 | 0.367 | 96(57-368) | 31(7-82) | 68(21-138) | 20(12-61) |
| 165 | CD8Tnaive | 0.432 | 319(131-334) | 134(43-282) | 128(60-212) | 64(31-333) |
| 166 | Treg | 0.006** | 657(137-1211) | 82(33-128) | 134(54-283) | 109(56-297) |
| 167 | CD4Tnaive | 0.056 | 163(23-518) | 24(12-193) | 70(34-176) | 60(12-148) |
| 168 | Th0 | 0.234 | 195(41-278) | 19(4-134) | 34(11-126) | 33(10-47) |
| 169 | CD4Tcm | 0.057 | 281(187-658) | 68(44-541) | 194(98-293) | 119(41-196) |
| 170 | UC | 0.254 | 179(40-1084) | 118(8-314) | 30(17-567) | 34(16-210) |
| 171 | Treg | 0.079 | 112(46-189) | 55(14-99) | 66(13-124) | 60(18-117) |
| 172 | CD4Tem | 0.068 | 378(298-1839) | 112(32-1380) | 238(59-718) | 102(19-419) |
| 173 | Monocytes | 0.85 | 45(9-308) | 21(11-74) | 25(4-167) | 45(12-111) |
| 174 | Monocytes | 0.413 | 20(8-67) | 9(3-14) | 9(4-35) | 9(4-35) |
| 175 | DPT | 0.106 | 146(126-313) | 61(34-124) | 155(75-229) | 72(50-154) |
| 176 | Monocytes | 0.624 | 233(130-576) | 137(59-298) | 234(66-399) | 158(101-600) |
| 177 | DNT | 0.057 | 85(31-198) | 36(11-102) | 65(21-142) | 26(17-80) |
| 178 | Treg | 0.069 | 383(146-923) | 60(35-150) | 111(52-276) | 78(39-183) |
| 179 | CD4Tcm | 0.112 | 81(66-241) | 28(18-163) | 81(40-165) | 38(21-112) |
| 180 | CD8Tem | 0.413 | 61(14-164) | 26(7-63) | 39(13-89) | 23(20-93) |
| 181 | CD8Tnaive | 0.479 | 41(19-82) | 17(5-35) | 35(9-47) | 30(13-39) |
| 182 | DNT | 0.085 | 46(19-138) | 23(8-55) | 25(5-60) | 23(6-30) |
| 183 | NKT | 0.678 | 36(11-41) | 17(7-40) | 26(14-40) | 15(8-29) |
| 184 | CD8Tcm | 0.027* | 292(103-424) | 86(27-174) | 103(76-285) | 119(43-159) |
| 185 | CD4Tcm | 0.209 | 45(28-474) | 11(3-79) | 30(10-48) | 10(2-24) |
| 186 | Monocytes | 0.303 | 193(48-411) | 100(62-139) | 240(50-272) | 148(47-236) |
| 187 | CD8Tnaive | 0.144 | 109(83-432) | 45(12-106) | 68(38-162) | 62(16-198) |
| 188 | CD4Tcm | 0.086 | 97(78-171) | 29(15-193) | 60(38-140) | 66(13-126) |
| 189 | CD8Tem | 0.384 | 60(21-105) | 14(3-40) | 30(10-51) | 24(6-30) |
| 190 | Treg | 0.376 | 123(48-235) | 56(8-63) | 34(20-127) | 23(16-62) |
| 191 | B cells | 0.038* | 520(112-1190) | 165(37-329) | 172(79-420) | 167(66-267) |
| 192 | CD4Tnaive | 0.042* | 166(66-483) | 27(17-259) | 71(30-171) | 40(5-135) |
| 193 | CD4Tcm | 0.27 | 25(7-39) | 12(4-26) | 17(5-45) | 11(7-39) |
| 194 | Tc0 | 0.18 | 126(57-1016) | 51(5-441) | 49(16-424) | 21(11-137) |
| 195 | DPT | 0.022* | 67(39-249) | 28(11-50) | 47(12-109) | 12(7-44) |
| 196 | NKs | 0.78 | 13(11-23) | 12(3-17) | 14(5-34) | 5(4-20) |
| 197 | NKs | 0.777 | 10(4-12) | 7(1-13) | 8(3-17) | 11(3-13) |
| 198 | NKs | 0.628 | 33(25-67) | 17(9-57) | 30(9-56) | 40(30-85) |
| 199 | Th1 | 0.224 | 8(6-24) | 9(6-24) | 10(3-41) | 5(4-20) |
| 200 | B cells | 0.248 | 8(2-14) | 1(1-9) | 5(1-13) | 1(0-6) |

**TableS1 Alteration in Cell count of the 200 SPADE nodes among the four time points.**

The population were presented as median and interquartile range, and P value of repeated measurement ANOVA were also listed.

* P <0.05; ** P<0.01; ***P<0.001

UC: unrecognized cells

| Node | Cell Type | Marker | P value | Time Point 1 | Time Point 2 | Time Point 3 | Time Point 4 |
| --- | --- | --- | --- | --- | --- | --- | --- |
| 2 | Tc2 | CD28 | 0.035 | 0.2031(0.08826-0.2811) | 0.0556(0-0.2151) | 0.1414(0.04691-0.2074) | 0.05991(0-0.1297) |
| 11 | Monocytes | CTLA-4 | 0.026 | 1.176(0.9676-1.311) | 0.8422(0.6922-1.063) | 0.9747(0.6362-1.081) | 0.8411(0.6988-1.243) |
| 16 | UC | Fas | 0.023 | 0.5638(0.258-0.7346) | 0.7072(0.3946-1.212) | 0.7443(0.5121-0.9473) | 0.8102(0.4811-1.082) |
| 18 | CD4Tem | CD28 | 0.024 | 0.3423(0.1996-0.4267) | 0.1086(0.04919-0.273) | 0.3322(0.1033-0.394) | 0.2421(0-0.3362) |
| 19 | CD4Tcm | PD-1 | 0.013 | 0.8854(0.8592-0.9482) | 1.382(0.9058-1.523) | 0.942(0.6284-1.14) | 1.004(0.731-1.271) |
| 28 | Monocytes | CD28 | 0.049 | 0.4391(0.3367-0.514) | 0.2877(0.03976-0.5604) | 0.4384(0.1901-0.5236) | 0.2326(0.06057-0.4471) |
| 33 | CD4Tem | CD28 | 0.012 | 0.2626(0.1154-0.3608) | 0.07862(0-0.2405) | 0.1492(0.05484-0.2715) | 0.1538(0-0.1899) |
| 56 | CD8Tem | PD-1 | 0.031 | 0.3532(0.1949-0.5308) | 0.5698(0.3424-0.7457) | 0.2179(0.01787-0.3647) | 0.2556(0.1186-0.5857) |
| 57 | CD8Tnaive | CD28 | 0.027 | 0.4524(0.2548-0.5312) | 0.247(0.07734-0.4914) | 0.3422(0.1618-0.4289) | 0.4655(0.3253-0.5269) |
| 78 | DNT | CD28 | 0.046 | 0.4282(0.2006-0.5871) | 0.2623(0.06817-0.4004) | 0.3291(0.2179-0.4773) | 0.3014(0.09147-0.433) |
| 111 | B cells | Fas | 0.049 | 0.8737(0.5907-1.213) | 1.159(0.8175-1.359) | 1.045(0.8458-1.419) | 1.094(0.9215-1.598) |
| 122 | Tc0 | Fas | 0.019 | 0.9087(0.6009-1.032) | 1.34(0.8131-1.48) | 0.9995(0.6014-1.325) | 1.258(1.058-1.554) |
| 125 | B cells | Fas | 0.027 | 1.155(0.799-1.328) | 1.287(1.067-1.662) | 1.293(1.122-1.592) | 1.47(1.233-1.745) |
| 135 | CD4Tem | CD28 | 0.045 | 0.1712(0.002765-0.2241) | 0(0-0) | 0(0-0.06485) | 0(0-0.1195) |
| 136 | CD8Tnaive | CD28 | 0.044 | 0.2555(0.1842-0.2812) | 0.1031(0-0.3256) | 0.137(0.09448-0.2286) | 0.2292(0.1193-0.2808) |
| 138 | B cells | Fas | 0.014 | 0.6191(0.4072-0.7456) | 1.138(0.4971-1.542) | 1.054(0.6523-1.218) | 0.8638(0.5838-1.215) |
| 154 | CD8Tem | Fas | 0.015 | 0.3689(0.2144-0.5468) | 0.7203(0.3287-1.027) | 0.4009(0.3356-0.8572) | 0.6112(0.3307-0.8444) |
| 157 | Monocytes | CTLA-4 | 0.034 | 0.96(0.7061-1.157) | 0.8758(0.7291-1.108) | 0.92(0.6318-1.131) | 0.6415(0.5454-1.017) |
| 167 | CD4Tnaive | ICOS | 0.049 | 0(0-0) | 0(0-0.01735) | 0(0-0) | 0.07845(0-0.2207) |
| 167 | CD4Tnaive | PD-1 | 0.022 | 0.2903(0.1739-0.3504) | 0.2113(0.1833-0.2968) | 0.4505(0.2517-0.555) | 0.357(0.273-0.4528) |
| 175 | DPT | CD28 | 0.047 | 0.4833(0.2285-0.6446) | 0.1939(0.07722-0.3762) | 0.3145(0.224-0.4901) | 0.197(0.1129-0.4394) |
| 177 | DNT | CXCR3 | 0.028 | 3.381(3.187-3.608) | 3.195(2.742-3.464) | 3.344(3.098-3.526) | 3.342(2.99-3.627) |
| 190 | Treg | PD-1 | 0.030 | 0.3993(0.1166-0.5128) | 0.2517(0-0.2633) | 0.3332(0.253-0.6013) | 0.4032(0.2531-0.4451) |
| 193 | CD4Tcm | PD-1 | 0.027 | 0.5959(0.06767-1.408) | 0.2232(0.05243-0.6874) | 0.049(0-0.5885) | 0.1011(0-0.4665) |
| 197 | NKs | OX40 | 0.035 | 3.868(2.473-4.375) | 2.481(0.1404-3.356) | 2.92(1.677-3.623) | 3.008(2.761-3.839) |
| 197 | NKs | ICOS | 0.046 | 0(0-0.974) | 0(0-0) | 0(0-0.1324) | 0(0-0.2103) |

**Table S2 SPADE nodes with significant altered functional markers and their marker expression.**

The marker expression were presented as median and interquartile range, and P value of repeated measurement ANOVA were also listed.

UC: unrecognized cells

| Term | Count | P value | Benjamini  Adjusted P value | Genes |  |
| --- | --- | --- | --- | --- | --- |
| Hepatitis B | 5 | <0.001 | 0.020 | NRAS, STAT5A, STAT1, PTEN, STAT2 | |
| Herpes simplex infection | 5 | 0.001 | 0.024 | HLA-DQB1, HLA-C, CCL5, STAT1, STAT2 | |
| Chemokine signaling pathway | 5 | 0.001 | 0.017 | NRAS, CCL5, STAT1, CCL4, STAT2 | |
| HTLV-I infection | 5 | 0.002 | 0.042 | HLA-DQB1, NRAS, STAT5A, IL2RG, HLA-C | |
| Natural killer cell mediated cytotoxicity | 4 | 0.002 | 0.039 | CD48, NRAS, CD244, GZMB | |
| Measles | 4 | 0.003 | 0.036 | STAT5A, IL2RG, STAT1, STAT2 | |
| Cell adhesion molecules | 4 | 0.003 | 0.034 | HLA-DQB1, CD8A, CD2, HLA-C | |
| Jak-STAT signaling pathway | 4 | 0.003 | 0.032 | STAT5A, IL2RG, STAT1, STAT2 | |
| Influenza A | 4 | 0.006 | 0.041 | HLA-DQB1, CCL5, STAT1, STAT2 | |
| Pathways in cancer | 4 | <0.001 | 0.020 | NRAS, STAT5A, STAT1, PTEN | |

**Table S3: Top 10 Enriched pathways**

Genes with significantly up-regulated expressions were analyzed using DAVID software to identify the significantly enriched pathways.
